# Optical Simulation-Aided Design and Engineering of Monolithic Perovskite/Silicon Tandem Solar Cells

**DOI:** 10.1021/acsaem.3c00136

**Published:** 2023-05-03

**Authors:** Yifeng Zhao, Kunal Datta, Nga Phung, Andrea E. A. Bracesco, Valerio Zardetto, Giulia Paggiaro, Hanchen Liu, Mohua Fardousi, Rudi Santbergen, Paul Procel Moya, Can Han, Guangtao Yang, Junke Wang, Dong Zhang, Bas T. van Gorkom, Tom P. A. van der Pol, Michael Verhage, Martijn M. Wienk, Wilhelmus M. M. Kessels, Arthur Weeber, Miro Zeman, Luana Mazzarella, Mariadriana Creatore, René A. J. Janssen, Olindo Isabella

**Affiliations:** † Photovoltaic Materials and Devices Group, Delft University of Technology, Partner in Solliance, 2628 CD Delft, The Netherlands; ‡ Molecular Materials and Nanosystems, Institute for Complex Molecular Systems, Eindhoven University of Technology, Partner in Solliance, P.O. Box 513, 5600 MB Eindhoven, The Netherlands; § Department of Applied Physics and Science of Education, Eindhoven University of Technology, Partner in Solliance, P.O. Box 513, 5600 MB Eindhoven, The Netherlands; ∥ TNO, Partner in Solliance, 5656 AE Eindhoven, The Netherlands; ⊥ TNO Energy Transition—Solar Energy, P.O. Box 15, 1755 ZG Petten, The Netherlands; # Eindhoven Institute for Renewable Energy Systems, P.O. Box 513, 5600 MB Eindhoven, The Netherlands; ¶ Dutch Institute for Fundamental Energy Research, 5612 AJ Eindhoven, The Netherlands

**Keywords:** perovskite, silicon heterojunction, tandem solar cells, optical simulations, two-terminal

## Abstract

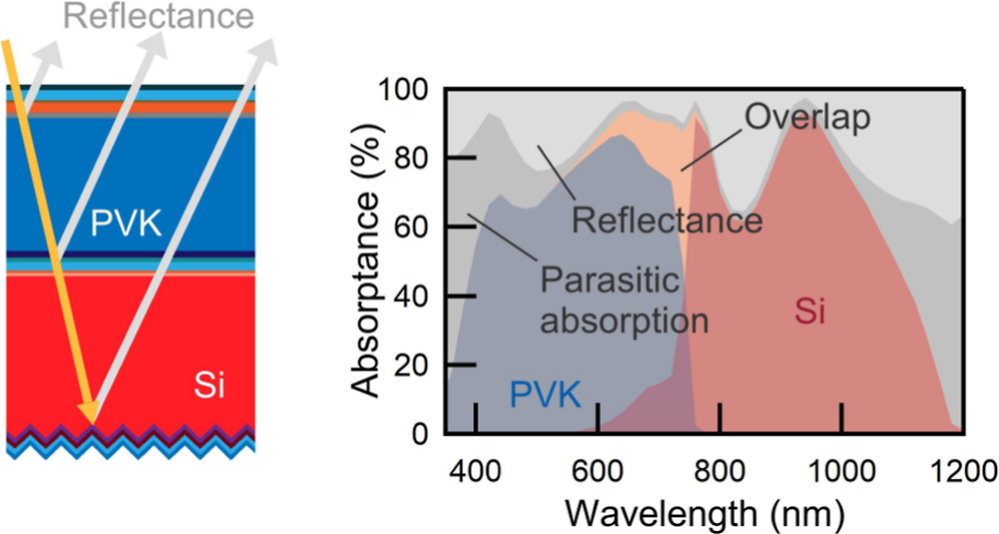

Monolithic perovskite/c-Si tandem solar cells have attracted enormous research attention and have achieved efficiencies above 30%. This work describes the development of monolithic tandem solar cells based on silicon heterojunction (SHJ) bottom- and perovskite top-cells and highlights light management techniques assisted by optical simulation. We first engineered (*i*)a-Si:H passivating layers for (100)-oriented flat c-Si surfaces and combined them with various (*n*)a-Si:H, (*n*)nc-Si:H, and (*n*)nc-SiO_*x*_:H interfacial layers for SHJ bottom-cells. In a symmetrical configuration, a long minority carrier lifetime of 16.9 ms was achieved when combining (*i*)a-Si:H bilayers with (*n*)nc-Si:H (extracted at the minority carrier density of 10^15^ cm^–3^). The perovskite sub-cell uses a photostable mixed-halide composition and surface passivation strategies to minimize energetic losses at charge-transport interfaces. This allows tandem efficiencies above 23% (a maximum of 24.6%) to be achieved using all three types of (*n*)-layers. Observations from experimentally prepared devices and optical simulations indicate that both (*n*)nc-SiO_*x*_:H and (*n*)nc-Si:H are promising for use in high-efficiency tandem solar cells. This is possible due to minimized reflection at the interfaces between the perovskite and SHJ sub-cells by optimized interference effects, demonstrating the applicability of such light management techniques to various tandem structures.

## 1 Introduction

The rapid development of monolithic two-terminal (2T) tandem solar cell technology combining perovskite and crystalline silicon (c-Si) sub-cells has led to significant increases in device performance in recent years. For instance, compared to the first reported devices exhibiting power conversion efficiencies (PCE) of approx. 14% in 2015, material and device optimization strategies to minimize optical losses and maximize energetic yield have increased PCE to above 32% in 2022.^1−3^ The flexible optical bandgap (*E*_g_), high absorptivity, and high defect tolerance observed in perovskite semiconductors make them ideal candidates for the top-cell in such tandem devices. A wide-bandgap (WBG) perovskite top-cell, typically prepared using mixed-halide (iodide-bromide) compositions, is used to allow balanced light absorption in the two sub-cells. Mainly thanks to their high open-circuit voltage (*V*_oc_) and good near-infrared (NIR) response,^4,5^ SHJ solar cells are among the most promising photovoltaic (PV) technologies that can be used as the bottom-cell in tandem devices.

High-efficiency tandem solar cells must feature a high *V*_oc_ that results from minimal non-radiative recombination losses in sub-cells. The key to obtaining a high *V*_oc_ in the bottom-cell mainly comes from excellent passivation of the c-Si surface by (*i*)a-Si:H.^6^ Therefore, optimization of (*i*)a-Si:H passivation is a prominent research focus for efficient SHJ solar cells. Similarly, a high radiative yield in iodide-based perovskite semiconductors typically enables a high *V*_oc_. However, mixed iodide-bromide perovskite compositions often suffer from higher non-radiative recombination,^7,8^ causing energetic losses in tandem devices. Strategies such as interfacial passivation or additive engineering have been shown to successfully suppress non-radiative recombination in the bulk of the perovskite layer and at interfaces with charge-selective contacts.^9,10^ Balancing carrier densities between sub-cells and maintaining high conductance through the recombination junction are additionally important to a successful integration of monolithic multijunction devices.^11,12^

Furthermore, in a 2T tandem device, balanced absorption of incident visible (vis) and NIR photons guarantees current matching conditions between the series-connected sub-cells and is critical to the high short-circuit current density (*J*_sc_) output of the multijunction solar cell. Barring that, the sub-cell generating the lowest current limits the overall output of the tandem device. As a result, light management guided by advanced optical simulations to minimize parasitic absorption and reflection losses is of great importance for tandem device design.^13−16^ Especially the reflection loss, due to suboptimal refractive index matching at intermediate interfaces between the top- and bottom-cells, should be minimized to enhance light coupling into the bottom sub-cell.

Lastly, a tandem device design must also account for processing challenges, specifically related to the development of the conformally coated solution-processed perovskite top-cells. As a result, bottom-cells with flat front-side and textured rear-side surfaces, that is, single-side-textured, are chosen in this study as a compromise between processing considerations of the perovskite top-cell and light absorption in the SHJ bottom-cell.

In this study, we describe the development and integration of 2T tandem solar cells based on SHJ bottom- and WBG perovskite top-cells. First, various (*i*)a-Si:H passivation approaches using different (*n*)-type layers (a-Si:H, nc-Si:H, and nc-SiO_*x*_:H) are compared, leading to the development of efficient single-side-textured SHJ single-junction devices with good *V*_oc_ and FF. Second, the construction of a stable, WBG mixed-halide perovskite solar cell is described in which interfacial energetic losses are minimized by using a self-assembled hole-transport monolayer and by passivating the electron-transport interface to improve the *V*_oc_. Finally, guided by advanced optical simulations, tandem solar cells with 1 cm^2^ active areas are fabricated by combining perovskite and SHJ sub-cells with minimized current mismatches between the sub-cells. The addition of an atomic layer deposition (ALD)-processed NiO_*x*_ layer in the recombination junction reduces electrical shunting in the monolithic tandems. Based on optical simulations, the role of (*n*)-layers in efficient light management as a route to boost the performance of tandem devices is discussed.

## 2 Results and Discussion

### 2.1 Development of Single-Junction Single-Side-Textured SHJ Solar Cells

The passivation optimization on a (100)-oriented flat c-Si surface is challenging as it is known to be prone to detrimental epitaxial growth of (*i*)a-Si:H as compared to the (111)-oriented surface,^17−21^ especially when a high hydrogen diluted (*i*)a-Si:H is used. Therefore, we study the passivation optimization for the (100)-oriented flat c-Si surface. In addition to optimizations of mono-(*i*)a-Si:H layers via tuning process conditions of plasma-enhanced chemical vapor deposition (namely, hydrogen dilution, pressure, power, substrate temperature), approaches such as bilayers and hydrogen plasma treatment (HPT) were also studied.^22−26^

**Figure 1:**
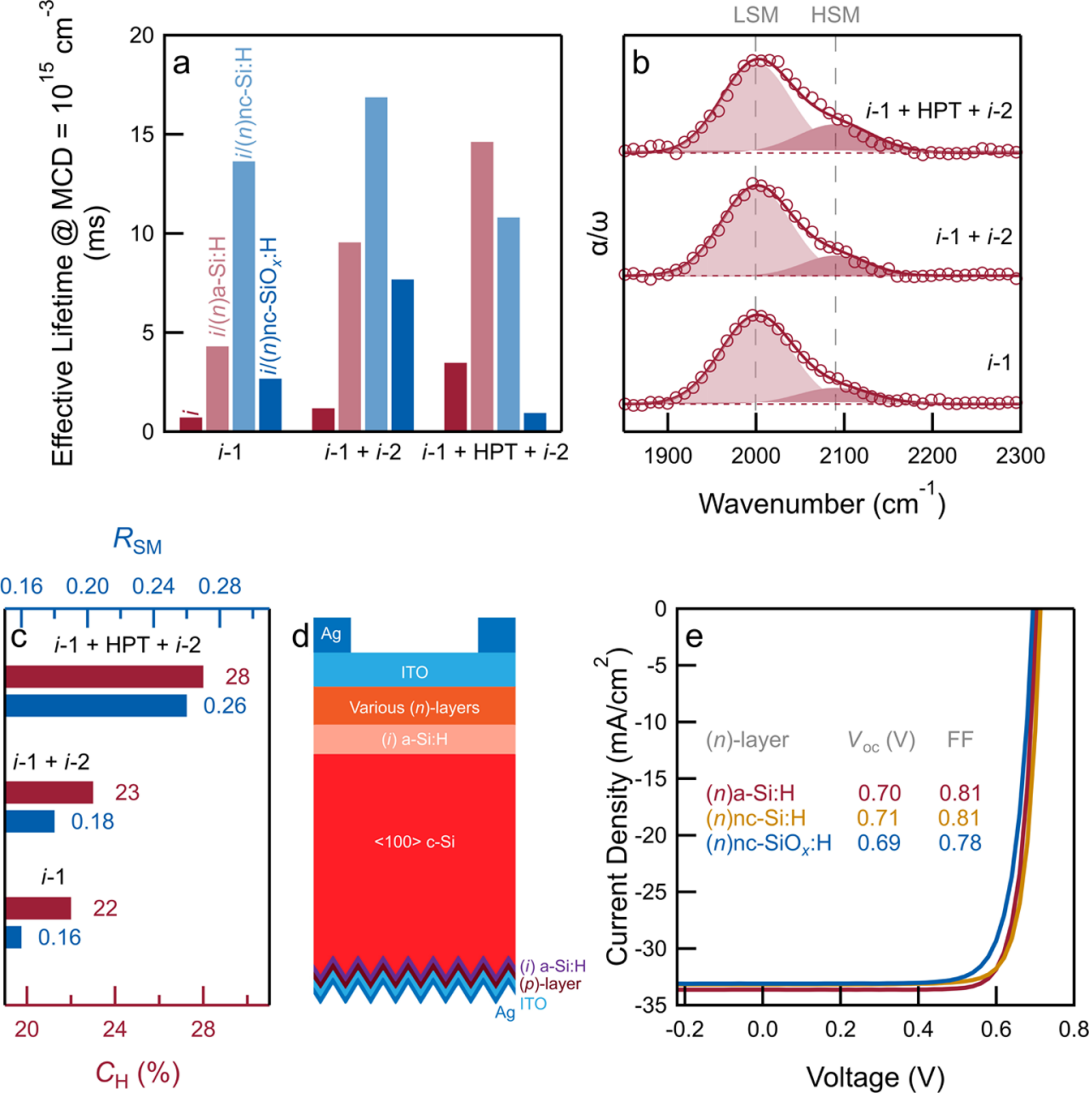
Passivation of single-side-textured SHJ solar cells based on (*n*)-type ⟨100⟩ wafers. (a) Effective lifetime (τ_eff_) of symmetrical, double-side-flat samples with different (*i*)a-Si:H passivation approaches (around 10 nm *i*-1, 5 nm *i*-1 + 5 nm *i*-2, 5 nm *i*-1 + HPT + 5 nm *i*-2) and different (*n*)-layers (a-Si:H, nc-Si:H, nc-SiO_*x*_:H). (b) Infrared spectra of various (*i*)a-Si:H layers (around 30 nm-thick). Empty circles represent measured data, solid lines represent overall fit, and shaded regions represent fitted Gaussian functions for LSM and HSM, where LSM and HSM stand for Si–H low- and high-frequency stretching modes, respectively. (c) Hydrogen content (*C*_H_) and microstructure factor (*R*_SM_) calculated from infrared spectra. (d,e) Schematic sketch (d) of single-junction single-side-textured rear junction SHJ solar cells with various front (*n*)-layers and their typical *J*–*V* curves (e).

Figure 1 shows the effect of passivation strategies on effective minority carrier lifetimes and the PV performance of single-junction solar cells. The deposition conditions and thicknesses/durations of the different passivation schemes are listed in Table S1 (Supporting Information). For samples with only *i* layer (Figure 1a), the effective lifetime (τ_eff_) can be increased from 0.7 ms for *i*-1 (prepared with only silane precursor) to 1.2 ms for a *i*-1 + *i*-2 bilayer (*i*-2 is prepared with the silane precursor and additional H_2_). Furthermore, by applying HPT at the interface between *i*-1 and *i*-2, the τ_eff_ is further enhanced to 3.5 ms. Infrared spectra (Figure 1b) indicate a higher absorption strength of the high-frequency stretching mode (HSM) when using a bilayer and/or the additional HPT. It is well-established that the low-frequency stretching mode (LSM) is assigned to monohydrides in small volume deficiencies,^27,28^ while HSM is mainly attributed to monohydrides and some polyhydrides at internal surfaces of larger volume deficiencies.^27−29^ As a result, higher hydrogen contents (*C*_H_) in the bilayer (*i*-1 + *i*-2) and the bilayer with HPT (*i*-1 + HPT + *i*-2) are calculated (Figure 1c). The increased absorption strength of HSM, and thus a higher microstructure factor (*R*_SM_), indicates that the film is more porous and more H-rich when we apply the bilayer, which further increases when using HPT.

The passivation is further improved when (*n*)-type layers are subsequently deposited on the *i*-layer (Figure 1a). When (*n*)a-Si:H is stacked on *i*-layer(s), noticeable τ_eff_ improvements are observed for all *i*-layer types. Following the τ_eff_ trend of *i*-layer only samples, we also observe optimized passivation quality and the highest absolute τ_eff_ improvement (τ_eff_ increased from 3.5 to 14.6 ms) when the bilayer with HPT was applied for (*n*)a-Si:H. This τ_eff_ improvement could be explained by their highest concentrations of HSM components for the bilayer with HPT, as seen in Figure 1b, which can further passivate the c-Si surface dangling bonds by acting as a H-reservoir for hydrogenations during the thermal processes.^30^ However, when using (*n*)nc-Si:H (τ_eff_ = 16.9 ms) and (*n*)nc-SiO_*x*_:H (τ_eff_ = 7.7 ms), the highest passivation is observed for the bilayer approach without the additional HPT. This is due to the fact that (*n*)nc-Si:H and (*n*)nc-SiO_*x*_:H require higher hydrogen dilution during their depositions as compared to (*n*)a-Si:H.^31^ As a result, the excessively incorporated hydrogen may induce additional defects near the c-Si surface,^32^ thus hindering the passivation quality. Similar to trends observed for nc-Si:H-based (*n*)-layers, bilayers without additional HPT also delivered optimized passivation quality for nc-Si:H-based (*p*)-layers [(*p*)nc-SiO_*x*_:H/(*p*)nc-Si:H] (Figure S1, Supporting Information). To summarize, although the bilayer without additional HPT does not exhibit the best (*i*)a-Si:H passivation, it relaxes the optimization efforts for further stacking on top, especially the high-hydrogen diluted doped layers.

Further, we applied different (*n*)-type layers combined with the bilayer (*i*-1 + *i*-2) to fabricate single-junction single-side-textured rear junction solar cells (Figure 1d). On the textured rear side of the solar cells, we applied the optimized contact stacks for hole collection.^33^ The τ_eff_, implied *V*_oc_, and implied fill factor of solar cells before metalization are provided in Table S2 (Supporting Information). Representative *J*–*V* characteristics (Figure 1e) show that both (*n*)a-Si:H and (*n*)nc-Si:H deliver *V*_oc_s above 0.70 V and FF approaching 0.81. Solar cells with (*n*)nc-SiO_*x*_:H exhibit slightly lower *V*_oc_ (0.69 V) and FF (0.78) than their non-oxidic counterparts. Nevertheless, in tandem devices, (*n*)nc-SiO_*x*_:H can present optical advantages by improving light coupling into the bottom-cell.^15^ The external quantum efficiency (EQE) spectra of these solar cells are presented in Figure S2 (Supporting Information). Single-junction cells using different (*n*)nc-SiO_*x*_:H thicknesses are shown in Figure S3 (Supporting Information).

### 2.2 Development of Single-Junction Mixed-Halide Perovskite Cells

**Figure 2:**
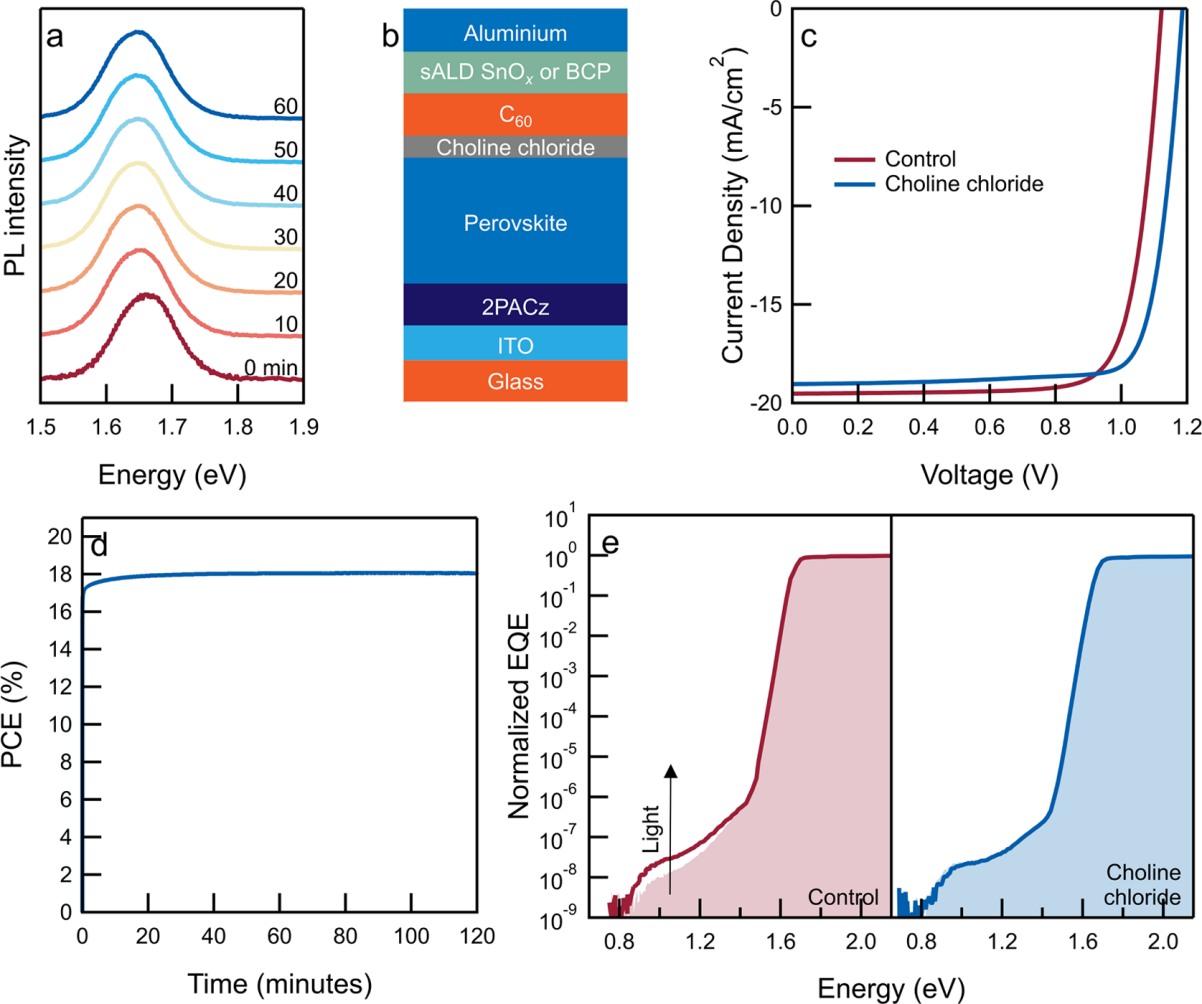
Stable, WBG perovskite solar cell. (a) Normalized photoluminescence spectra as a function of time under continuous illumination (λ = 405 nm, ∼4 Sun equivalent intensity) of the K_0.05_Cs_0.05_(FA_0.75_MA_0.25_)_0.90_Pb(I_0.75_Br_0.25_)_3_ perovskite film deposited on the glass substrate. The spectra are plotted on a linear intensity axis and offset vertically for clarity. (b) Device layout of a p-i-n single-junction perovskite solar cell. (c) Current density vs voltage scans of solar cells without (control) or with a choline chloride surface passivation layer. (d) PV PCE as a function of time of the choline chloride-treated solar cell at maximum power point voltage, *V*_MPP_ = 1.02 V. (e) Sub-bandgap photocurrent spectra of perovskite solar cells without (control) or with choline chloride surface treatment. In each device, the shaded spectra represent the pristine device, and the line represents the spectra after being illuminated with a 532 nm CW laser at ∼1 Sun equivalent intensity. The control device was illuminated for 1 h, whereas the choline chloride-treated device was illuminated for ∼16 h. The spectra of pristine cells are normalized to the bandgap onset, and the spectra of irradiated cells are plotted relative to those of pristine cells. sALD SnO_*x*_ was used in devices shown in (c,d), whereas evaporated BCP (8 nm) was used in devices shown in (e).

The perovskite absorber (nominal composition Cs_0.05_(FA_1–*x*_MA_*x*_)_0.95_Pb(I_1–*x*_Br_*x*_)_3_) was prepared using an antisolvent-based recipe that allowed changing the perovskite composition to alter the *E*_g_ of the layer, between 1.63 eV for a 17% Br-containing (*x* = 0.17) perovskite and 1.75 eV for a 33% Br-containing (*x* = 0.33) composition (Figure S4, Supporting Information). In comparison, perovskite layers used in most high-efficiency tandems have an *E*_g_ of about 1.68 eV to reduce current mismatch between sub-cells.^34^ Hence, a 25% Br-containing perovskite was chosen for subsequent experiments. Potassium was added to the perovskite precursor [nominal composition K_0.05_Cs_0.05_(FA_0.75_MA_0.25_)_0.90_Pb(I_0.75_Br_0.25_)_3_] to improve PV performance and reduce light-induced instabilities (Table S3 and Figures S5 and S6, Supporting Information).^9^ As a result, photoluminescence spectra (Figure 2a) recorded over 60 min of continuous illumination (405 nm, 4 Sun equivalent intensity) do not show a strong red shift of the emission peak as a result of light-induced halide segregation in mixed-halide perovskites. Instead, only a minor shift in the emission peak is observed (Figure S7, Supporting Information). Such an effect has previously been described to decrease the *J*_sc_ of solar cells.^35,36^ The high stability is likely due to the lower defect density that otherwise acts as an ion migration pathway, which drives such instability in thin films;^37,38^ this is also supported by the high photoluminescence lifetime (approx. 450 ns) of perovskite films (Figure S8, Supporting Information).

Using this perovskite composition, inverted (p-i-n) solar cells were developed using (2-(9*H*-carbazol-9-yl) ethyl) phosphonic acid (2PACz) hole-transport and C_60_ electron-transport layers. The choice of 2PACz stems from better energetic alignment between the valence band maximum of the perovskite and the highest occupied molecular orbital level of 2PACz compared to that of the commonly used polymeric transport layer, poly[bis(4-phenyl)(2,4,6-trimethylphenyl)amine] (Figure S9, Supporting Information).^34,39^ Ambient-pressure spatial ALD (sALD) was used to deposit a SnO_*x*_ layer between the C_60_ and the metal contact. In monolithic tandem devices, this layer acts as a buffer layer to protect against sputter damage during indium tin oxide (ITO) deposition.^40,41^ The thickness of the SnO_*x*_ layer was found to not significantly affect device performance (Figure S10, Supporting Information), and layers as thin as 6 nm were effective in both opaque and semi-transparent devices.^42^ Similar performances could also be achieved using temporal ALD, which is carried out in high-vacuum conditions (Figure S11, Supporting Information).^43^

The open-circuit voltage of p-i-n solar cells has been shown to be significantly limited by non-radiative recombination at the perovskite/C_60_ interface.^7,10,44^ Surface treatment using choline chloride was used to passivate the interface in order to minimize losses.^45^ Figure S12 (Supporting Information) shows X-ray photoelectron spectroscopy (XPS) data in the N 1s region of a bare and a treated perovskite layer and confirms the presence of quaternary ammonium species (binding energy ∼ 403 eV), related to the choline moiety. Surface morphology, characterized by scanning electron microscopy (SEM) (Figure S12, Supporting Information), also confirms the presence of amorphous domains on the surface, which we attribute to the presence of choline chloride (Figure S12b,c, Supporting Information). Absolute photoluminescence spectroscopy was used to characterize recombination processes at the perovskite/C_60_ interface, which reveals that non-radiative recombination is suppressed upon passivating the interface with choline chloride, leading to an increase in the photoluminescence intensity.^7,46^ As a result, while placing C_60_ on top of the perovskite results in a decrease of the quasi-Fermi level splitting by approx. 115 mV in the control sample, the loss is reduced to approx. 6 mV in the passivated sample (Figure S13, Supporting Information). In solar cells, the treatment results in a *V*_oc_ gain of 60 mV, amounting to an increase from 1.13 to 1.19 V (Figures 2c and S14, Supporting Information).

Solar cells also show no performance degradation due to light-induced instabilities under continuous operation at *V*_MPP_ for 2 h (Figure 2d), further confirming the stability of the perovskite layer against halide segregation. Sensitive photocurrent spectroscopy was then used to probe the contribution of sub-bandgap states and their response to continuous illumination.^36^ Previously, it has been shown that in unstable perovskite compositions, the photocurrent contribution of sub-bandgap states increases under continuous irradiation, along with the development of a low-energy shoulder at the band-edge that confirms the formation of iodide-rich domains.^36,47,48^ The spectra show (Figure 2e) that in the case of the control device (without choline chloride treatment), a small increase in the contribution from defect states occurs, indicating that defect states respond to irradiation (λ = 532 nm, ∼1 Sun equivalent intensity, 1 h). It must be noted that the response is far less severe compared to that observed in compositions that undergo severe halide segregation,^36^ indicating the innate stability of the perovskite layer. Nevertheless, in a choline chloride-treated solar cell, after ∼16 h of light exposure, the defect contribution across the sub-bandgap region remains unchanged, pointing to improved stability as a result of surface treatment (Figure S15, Supporting Information).

Semi-transparent solar cells using ITO to replace the Al metal contact show a decrease in PV performance on account of reduced light absorption due to the transmission of NIR light (Figure S16a, Supporting Information). However, no significant losses in *V*_oc_ occur, indicating a virtually lossless SnO_*x*_/ITO interface. There is a marginal decrease in the FF due to the higher sheet resistance of the ITO contact compared to the metal electrode. The EQE spectra (Figure S16b, Supporting Information) show a reduced response in the 400–500 nm range when the solar cell is illuminated via the electron-transport layer (C_60_) side, which is more representative of operation when integrated into a tandem cell, due to parasitic absorption in the C_60_ layer. The parasitic absorption can be decreased by reducing the C_60_ thickness from 20 to 10 nm.^42^ Reflection at the air/ITO interface and parasitic absorption in the ITO layer also reduce absorption in the perovskite layer and can be manipulated by changing the ITO layer thickness and through the addition of anti-reflective coatings such as MgF_2_.^14^

### 2.3 Integration of Monolithic Perovskite/SHJ Tandem Solar Cells

Tandem solar cells with an active area of 1 cm^2^ were prepared by combining the SHJ and perovskite sub-cells. Figure S17 (Supporting Information) shows an image of the cell along with cross-sectional SEM images of the perovskite top-cell and the textured rear interfaces of the SHJ bottom-cell. Here, the perovskite grains span the thickness of the film, leading to minimal recombination losses due to the presence of grain boundaries, which benefits the *V*_oc_ of the devices.

However, using only 2PACz in the recombination junction together with ITO yields unsatisfactory results where all of the top-cells are shunted. Hence, an ALD-processed NiO_*x*_ layer was used in the recombination junction to reduce electrical shunting.^49^ Previously, such layers have also been shown to benefit performance yield and reproducibility in perovskite single-junction and perovskite-based multijunction devices.^42,50^ Increasing lateral resistance at the interface, such as through an undoped NiO_*x*_ as in this case, has also been shown to benefit tandem device performance.^51^ We used electrostatic force microscopy (EFM) to characterize the distribution of 2PACz on ITO or ITO/NiO_*x*_ substrates (Figure S18, Supporting Information). No discernible differences could be observed in the contact potential difference (CPD) maps. In particular, the probability distribution curves of the CPD for both cases have a similar full width at half maximum (FWHM) of around 7.9 and 9.3 mV (ITO or ITO/NiO_*x*_ with 2PACz layers, respectively). This suggests that the molecular distribution of 2PACz is similar on both substrates.

We employed transmission electron microscopy (TEM) characterization of tandem devices to examine the ITO/NiO_*x*_/2PACz/perovskite interface (Figure S19, Supporting Information). The 2PACz layer, which is rich in carbon, appears as a bright, thin layer on ITO or NiO_*x*_. Although continuous 2PACz layers can be detected in both ITO/2PACz- and ITO/NiO_*x*_/2PACz-based tandem devices, corroborating the similar 2PACz distribution observed in EFM (Figure S18, Supporting Information), the thickness of 2PACz on ITO is clearly lower than on NiO_*x*_/ITO (ca. 2 vs 5 nm). We believe that the ultrathin 2PACz on ITO is prone to shunting when it is implemented in devices, which leads to non-working tandem devices.^49^ In contrast, the presence of hydroxyl-rich NiO_*x*_ facilitates the assembly of 2PACz, resulting in a reduction of electrical shunts in the top-cells.

**Figure 3:**
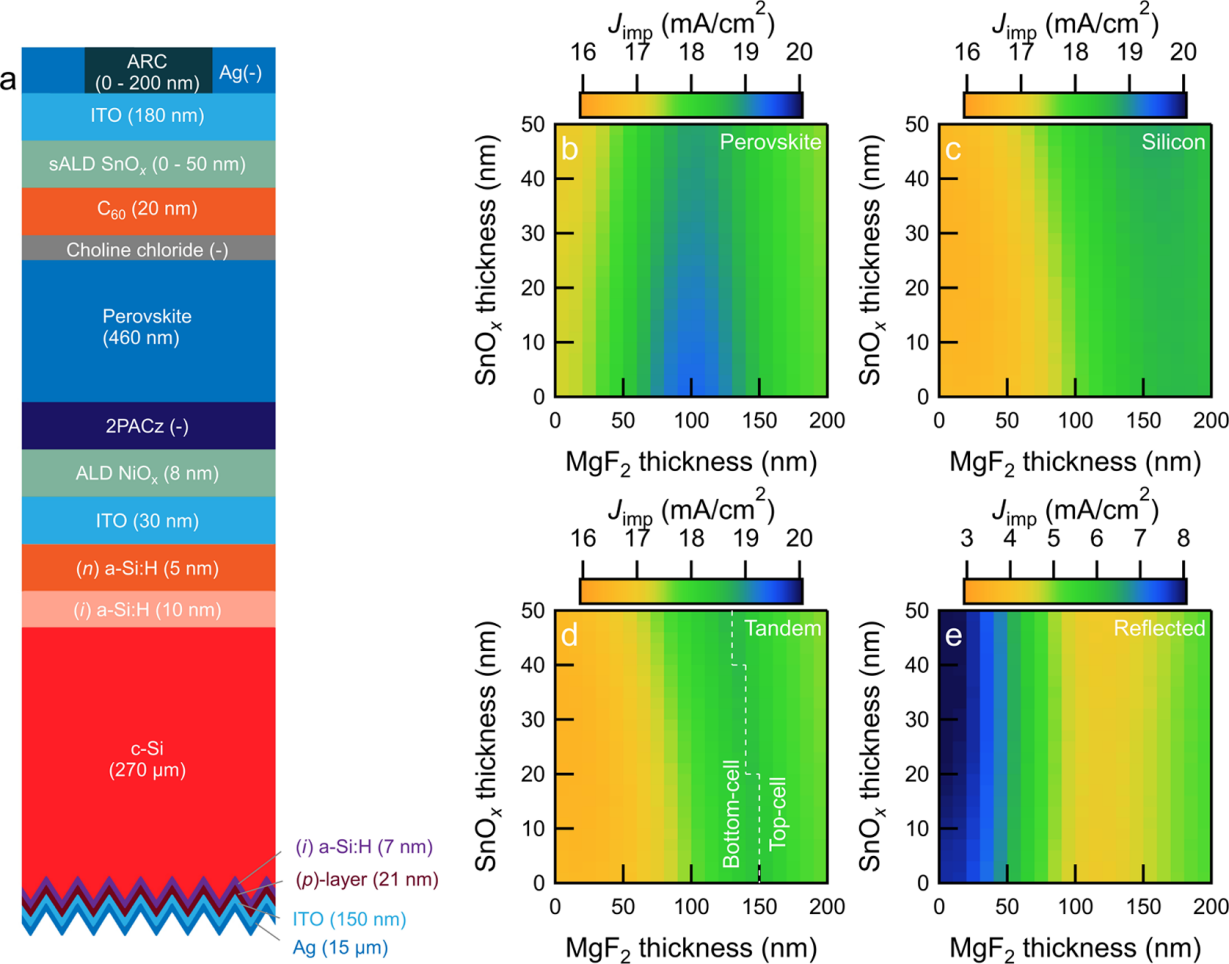
Optical simulations of perovskite/SHJ tandem devices. (a) Schematic sketch of the perovskite/SHJ solar cell used for simulations. (b–e) Implied photocurrent density in (b) perovskite top-cell, (c) silicon bottom-cell, (d) tandem cell, and (e) implied reflected photocurrent density. The dashed line in (d) distinguishes between configurations that are limited either by the bottom-cell or by the top-cell.

**Figure 4:**
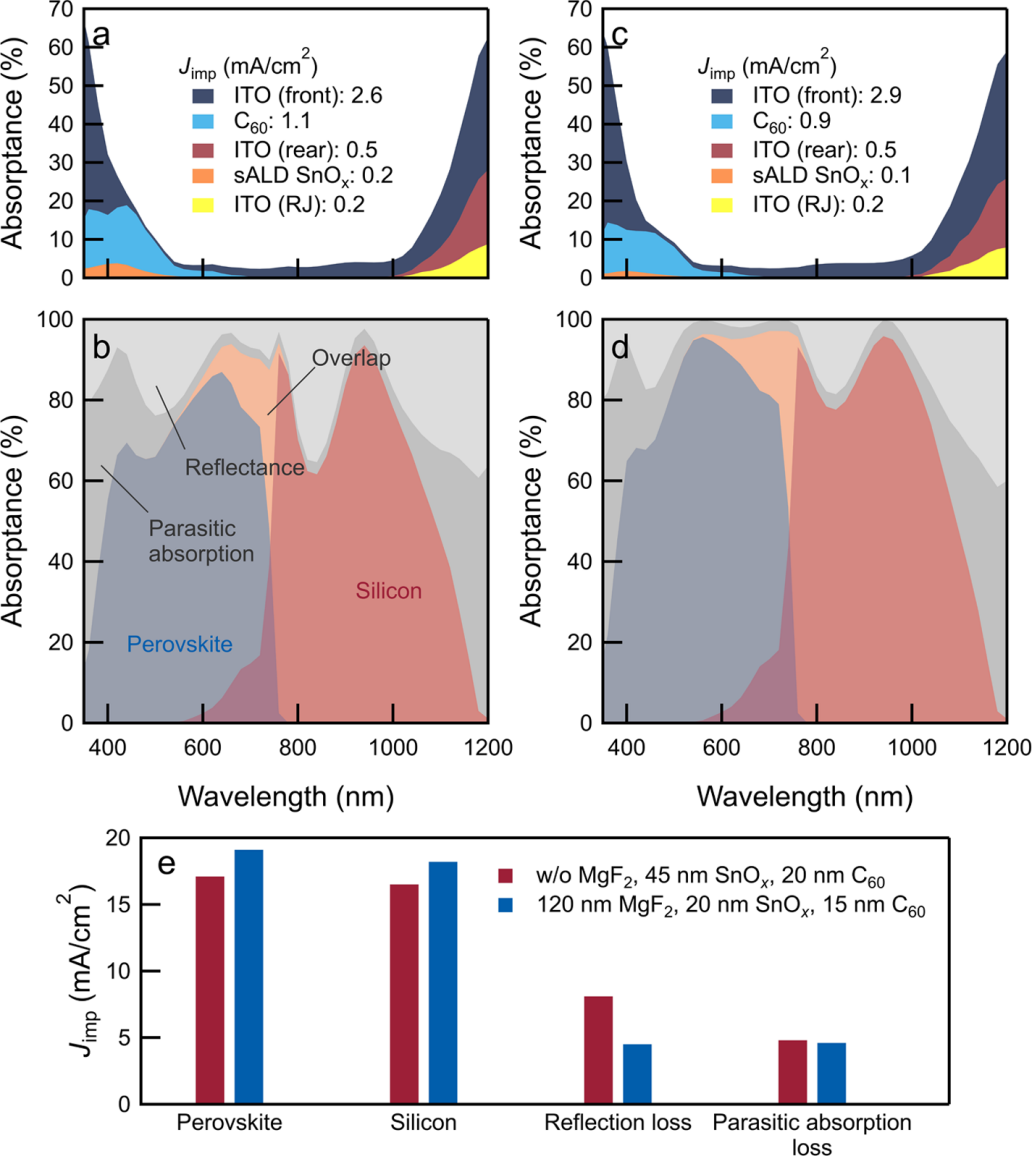
Simulated absorptance spectra of the cells (a,b) without MgF_2_ and a thick (20 nm) C_60_ layer and (c,d) with MgF_2_ (120 nm) and a thin (15 nm) C_60_ layer. The SnO_*x*_ thickness is 45 nm (20 nm) before (after) the optimizations, respectively. In (b,d), the orange area represents the sum of the absorptance of the perovskite and silicon sub-cells. (e) Integrated *J*_imp_ for both sub-cells, reflection, and parasitic absorption losses before and after optimizations.

Optimized light management in perovskite/SHJ tandem solar cells is important for achieving high current densities at current-matching conditions. Therefore, using the GenPro4 simulation tool,^52^ we performed optical simulation studies based on our practical tandem devices to further guide the experimental work. The optical constants of all layers used in the top-cell and all thin-film Si layers and ITO used in the bottom-cell were obtained from spectroscopic ellipsometry measurements. The schematic structure of the monolithic perovskite/SHJ tandem solar cell with (*n*)a-Si:H as a representative is illustrated in Figure 3a. Considering one electron–hole pair generated by each absorbed photon, negligible recombination, and only the active area (without front metal electrodes), the simulated implied photocurrent density (*J*_imp_) of sub-cells, tandem cells, and the reflected fraction in the wavelength range from 300 to 1200 nm are shown in Figure 3b–e. Figure 4 shows the simulated absorption in different layers of the stack as a function of layer thickness.

By identifying the minima in implied reflected photocurrent density and the maxima in implied photocurrent density contributions of the sub-cells, optimum thicknesses of MgF_2_ and SnO_*x*_ layers were found. The highest implied photocurrent density in the perovskite top-cell (Figure 3b) was found at a MgF_2_ thickness of around 100 nm, whereas for the SHJ bottom-cell (Figure 3c), the thickness was between 160 and 180 nm. The implied photocurrent density of the tandem device (Figure 3d) was determined by the current-limiting sub-cell, which is related to both the reflection losses (Figure 3e) and the absorption characteristics of both sub-cells. It was found that the maximum implied tandem current density (approximately 18.6 mA/cm^2^) requires a MgF_2_ layer with a thickness of approximately 120–160 nm, whereas the thickness of SnO_*x*_ is of minor consequence.

Figure 4 further shows the influence of layer thicknesses (C_60_, SnO_*x*_, and MgF_2_) on the simulated spectral response of the tandem device. Two sets of values for C_60_, SnO_*x*_, and MgF_2_ thicknesses were considered (20, 45, 0 and 15, 20, and 120 nm, respectively) to illustrate changes in reflection and parasitic absorption. The absence of an anti-reflective coating leads to reflection losses in the 400–500 nm range (Figure 4b,d), decreasing absorption in the perovskite top-cell; reflection loss also leads to a minimum in the 800–900 nm range, which reduces absorption in the silicon bottom-cell. Parasitic absorption in the C_60_ layer (400–500 nm) further decreases absorption in the perovskite sub-cell.^42^

By decreasing the C_60_ and SnO_*x*_ layer thickness (Figure 4d) and introducing the anti-reflective coating, reflection losses are minimized by an equivalent of 3.6 mA/cm^2^ (see Figure 4e). This increase in spectral response agrees with experimental observations (Figure S20, Supporting Information) where the EQE dip at ∼850 nm flattens with increasing MgF_2_ thickness. At the same time, the response of the top-cell in the 550–700 nm range increases, leading to an overall improvement in total absorbed light from an equivalent of 32.1–35.4 mA/cm^2^ with an optimum at approximately 120 nm-thick MgF_2_ (Figure S20b, Supporting Information). Figure S21 (Supporting Information) further shows that reducing the C_60_ layer thickness from 20 to 10 nm yields an increase of 0.9 mA/cm^2^ in the top-cell *J*_sc_ contribution by reducing parasitic absorption. Reflection losses that influence the response of both sub-cells can also be reduced by altering the thickness of the SnO_*x*_ layer. For example, as also seen in Figure S21a, reducing the thickness from 45 to 6 nm causes reflection losses to be minimized in the 800–900 nm range. However, this is countered by a decrease in absorption at ∼1000 nm, which collectively results in only a marginal 0.3 mA/cm^2^ gain in the *J*_sc_ contribution of the SHJ sub-cell. Figure S22 (Supporting Information) further compares experimentally determined EQE spectra to simulated absorptance spectra and finds good agreement between the behavior of devices using different MgF_2_ and C_60_ layer thicknesses.

**Figure 5:**
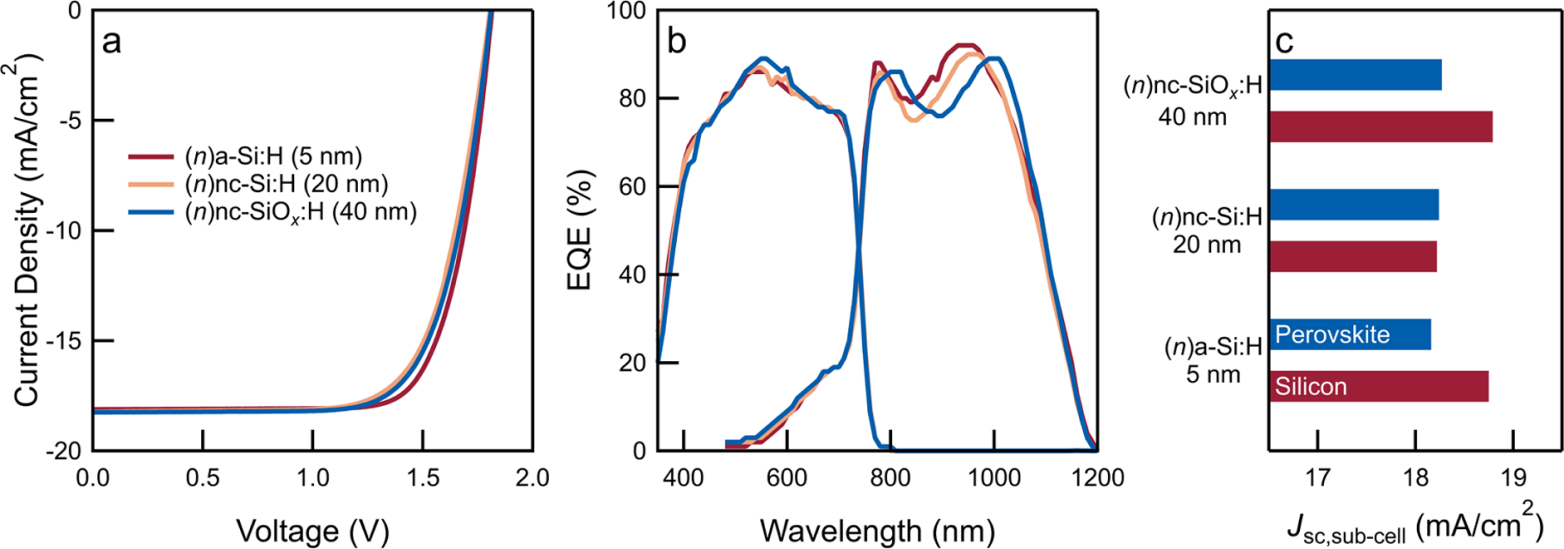
PV performance of optimized tandem solar cells. (a) *J*–*V* curves and (b) EQE spectra of monolithic tandems prepared using different (*n*)-type layers (5 nm (*n*)a-Si:H, 20 nm (*n*)nc-Si:H, and 40 nm (*n*)nc-SiO_*x*_:H). (c) *J*_sc_-contributions of top- and bottom-cells extracted from EQE spectra (b).

In all cases, a large current mismatch (>1 mA/cm^2^) is found to decrease the *J*_sc_ of the tandem solar cell, with the perovskite top-cell being the current limiting factor. As a result, by decreasing the bromide content in the perovskite from 25 to 21%, thereby narrowing the optical bandgap from 1.69 to 1.67 eV,^53^ the top-cell contribution to *J*_sc_ can be improved. Table 1 and Figure 5 show the PV performance of tandem solar cells using different (*n*)-type layers described in Section 2.1. The (*n*)-type layers predominantly influence optical interference in the NIR region that affects *J*_sc,silicon_. For instance, using a 20 nm-thick (*n*)nc-Si:H layer leads to a current matched tandem solar cell at a *J*_sc_ of 18.2 mA/cm^2^. On the other hand, using a 5 nm-thick (*n*)a-Si:H or 40 nm-thick (*n*)nc-SiO_*x*_:H layer causes a slightly higher absorption in the bottom-cell, whereas the top-cell response remains largely unchanged, leading to top-cell-limited tandem devices.

**Table 1:** PV Performance of Single-Junction Perovskite and SHJ Solar Cells and Monolithic Tandem Devices^a^

solar cell type	*V*_oc_ (V)	*J*_sc_ (mA/cm^2^)	FF	PCE (%)	PCE gain* (SHJ) (abs. %)	PCE gain^#^ (perovskite) (abs. %)
Single-junction Solar Cells						
perovskite	1.15	20.2	0.79	18.3		
SHJ (*n*)a-Si:H	0.70	33.6	0.81	19.1		
SHJ (*n*)nc-Si:H	0.71	33.2	0.81	19.1		
SHJ (*n*)nc-SiO_*x*_:H	0.69	33.1	0.78	17.8		
Tandem Solar Cells						
(*n*)a-Si:H	1.81	18.1	0.75	24.6	5.5	6.3
(*n*)nc-Si:H	1.81	18.2	0.71	23.4	4.3	5.1
(*n*)nc-SiO_*x*_:H	1.81	18.3	0.70	23.2	5.4	4.9

a * and ^#^ indicate the absolute gain in PCE in tandem solar cells compared to the PCEs of corresponding SHJ and perovskite single-junction solar cells, respectively.

In all cases, a high *V*_oc_ of 1.81 V can be measured, representing a low *V*_oc_ loss (30–50 mV) upon integration compared to the sums of the *V*_oc_s of perovskite and SHJ single-junction devices. A proportion of the *V*_oc_ loss results from reduced light incidence on the SHJ sub-cell, while additional interfacial losses at the recombination junction can also lower the *V*_oc_. Finally, on account of a higher FF (75%), the cell based on the (*n*)a-Si:H layer yields a tandem PCE of 24.6% compared to cells based on (*n*)nc-Si:H (23.3%) or (*n*)nc-SiO_*x*_:H (23.7%), representing a 5.5% absolute gain from the single-junction PCE of the SHJ solar cell and a 6.3% absolute gain from that of the perovskite solar cell.

### 2.4 Discussion and Outlook

To further explore the potential of using different (*n*)-layers in tandem solar cells, we identified optimum (*n*)-layer thicknesses through optical simulations. To better understand the origin of reflection losses in simulated tandem solar cells, we decomposed the total front reflection into three components: reflections at the front of the perovskite top-cell (*R*_1_), reflections in between the perovskite top-cell and SHJ bottom-cell (*R*_2_), and reflections at the rear side of the SHJ bottom-cell (*R*_3_).^16^ The refractive indices of different (*n*)-layers can be found in Figure S23 (Supporting Information). Before simulating, we checked the simulation model by comparing the simulated absorption with the measured EQE curves reported in Figure 5b (Figure S24, Supporting Information), which show good correspondence between the simulation and measured data.

**Figure 6:**
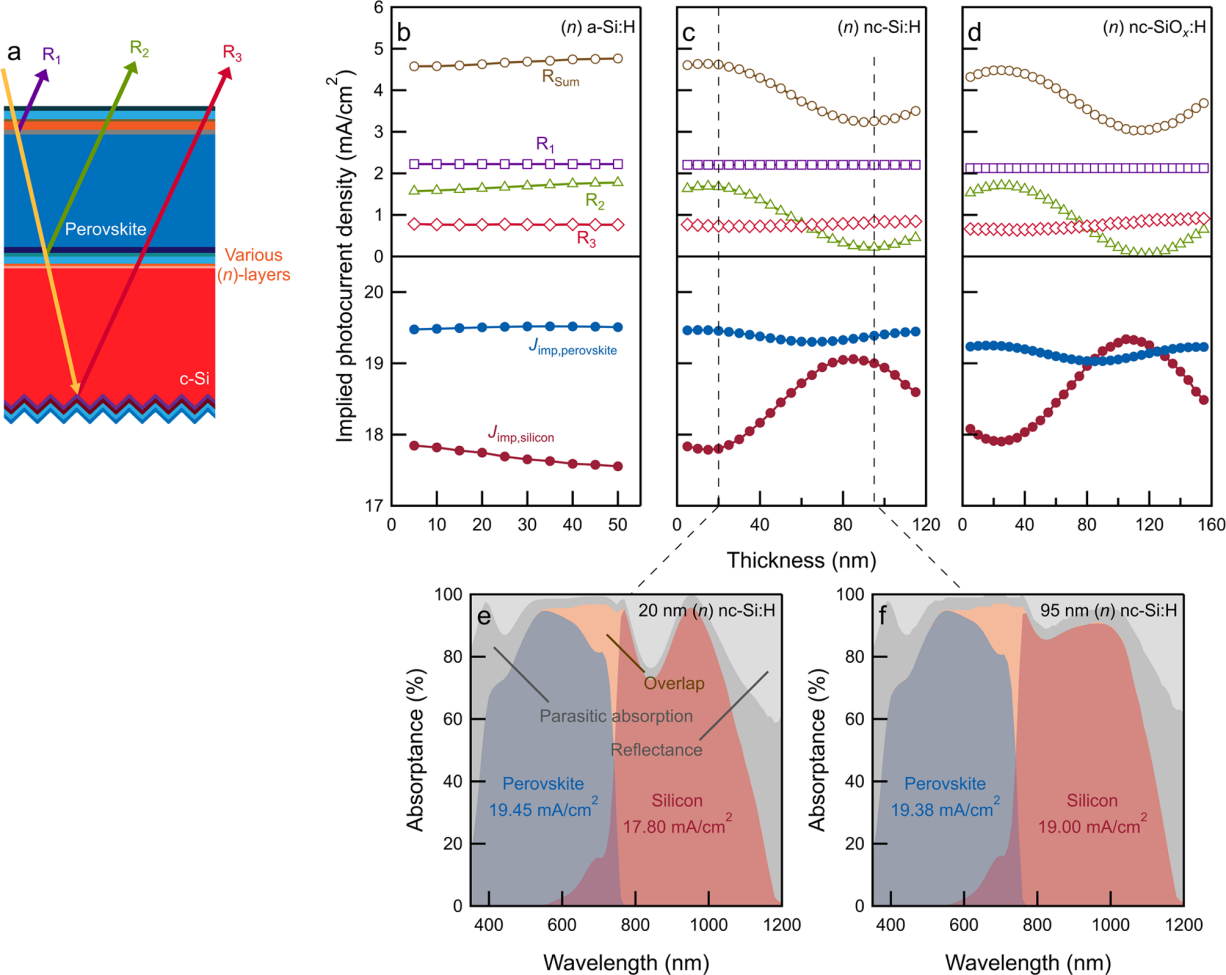
Light management in monolithic tandem solar cells. (a) Schematic of the perovskite/SHJ tandem solar cell for optical simulations and (b–d) implied photocurrent density of the perovskite top-cell, SHJ bottom-cell, and reflected light as a function of (*n*)-layer thickness and types of (*n*)-layer. (e,f) Simulated absorptance spectra of tandem solar cells using (*n*)nc-Si:H interlayers with a thickness of (e) 20 and (f) 95 nm.

Figure 6 shows the implied photocurrent density in sub-cells and tracks the reflected light as a function of (*n*)-layer thickness. In all cases, *R*_1_ and *R*_3_ are nearly constant across all configurations using different (*n*)-layer thicknesses, whereas *R*_2_ is strongly influenced by the (*n*)-layer. The optimum thickness of (*n*)a-Si:H is 5 nm (Figure 6b); further increase of the (*n*)a-Si:H thickness results in increased internal reflections between the top- and bottom-cells (*R*_2_), which reduces the amount of light being coupled into the SHJ bottom-cell, widening the current mismatch between sub-cells. In contrast, the *R*_2_ is minimized when the thicknesses of (*n*)nc-Si:H (Figure 6c) and (*n*)nc-SiO_*x*_:H (Figure 6d) are optimized to 95 and 115 nm, respectively. Especially in this case, with (*n*)nc-SiO_*x*_:H, we can minimize *R*_2_ to below 0.1 mA/cm^2^. Electrically, as demonstrated in Figure S3, SHJ solar cells using a thick (around 110 nm-thick) (*n*)nc-SiO_*x*_:H layer can still exhibit an average FF approaching 79%, demonstrating its potential for the high-efficiency tandem solar cell.^1,15^ Similarly, we expect the (*n*)nc-Si:H layer to be a promising candidate for high-efficiency tandem devices.^54^ The observed beneficial effects upon thickness optimization of (*n*)nc-SiO_*x*_:H and (*n*)nc-Si:H can be attributed to their better refractive index matching in the tandem solar cell than (*n*)a-Si:H (Figure S23, Supporting Information).

For example, taking the case of (*n*)nc-Si:H, Figure 6e,f shows the simulated absorptance spectra of tandem solar cells with 20 and 95 nm (*n*)nc-Si:H interlayers. The parasitic absorption induced by (*n*)nc-Si:H is increased from 0 to 0.3 mA/cm^2^ (Figure S25, Supporting Information) when the thickness of (*n*)nc-Si:H is increased from 20 to 95 nm. Nevertheless, Figure 6e,f reveals that the reflection loss in the 800–900 nm wavelength range, which is primarily caused by the intermediate interfaces, is significantly suppressed. This decrease in reflection loss, as also seen in Figure 6c, where *R*_2_ is reduced from 1.7 to 0.2 mA/cm^2^, is substantial enough to outweigh the additional parasitic absorption loss. Therefore, the implied photocurrent density of the c-Si bottom-cell increased from 17.8 to 19.0 mA/cm^2^. The absorptance in other layers remains nearly unaffected. The observation of reduced *R*_2_ at intermediate interfaces can be explained by the interference effect, which can be affected by the wavelength-dependent refractive indices [e.g., different types of (*n*)-layers], and thicknesses of layers used in the device. As a proven first-order approximation,^15^ the advantageous implementation of (*n*)nc-Si:H and (*n*)nc-SiO_*x*_:H as seen in Figure 6c,d can be attributed to their proper refractive indices that lie between those of perovskite and c-Si absorbers within the 800–1200 nm wavelength range. In contrast, the refractive indices of (*n*)a-Si:H do not exhibit such a favorable match (Figure S23, Supporting Information). Additionally, by adjusting the optical thickness, which is the product of the refractive index and the film thickness, destructive interference can be achieved, leading to increased light transmittance to the bottom-cell.

Optimizing interference effects, especially due to intermediate interfaces (*R*_2_), as a route to light management in tandem devices can therefore be a general strategy to optimize device design for maximizing matched tandem current density. Similarly, the strategy used here for front-side-flat tandem solar cells can also be applied to optimize double-side-textured (typical pyramidal texture) tandem devices, as demonstrated in Figure S26 (Supporting Information). However, the benefits of using (*n*)nc-Si:H or (*n*)nc-SiO_*x*_:H over (*n*)a-Si:H are less significant in double-side-textured devices, as textured surfaces already result in decreased reflection losses.

On the other hand, the *V*_oc_ of tandem devices is restricted by the *V*_oc_ of the perovskite sub-cell due to non-radiative recombination in WBG compositions.^7,8^ Recent reports have described the use of compositional variations and additives to minimize bulk defects as a route to increase the radiative yield.^55,56^ At the same time, interfacial losses also contribute to *V*_oc_ losses.^57,58^ The recent development of SAM-based hole-transport layers shows that the *V*_oc_ and FF of solar cells can be significantly improved by better energetic alignment and fast hole extraction, which can also improve device stability against light-induced halide segregation.^34,39,59−62^ The interface with the electron-transport layer, C_60_, is also crucial in WBG devices due to increased non-radiative recombination arising from this interface with widening the optical bandgap.^7,63^ By improving electron-selective contact layers,^64,65^ and through interfacial treatments such as with choline chloride (used in our study), lithium fluoride, or lower-dimensional perovskites, energetic losses at that interface can be suppressed.^7,45,66^ The *V*_oc_ of the bottom SHJ cell can also be further improved by improving the processing of contact layers. Especially due to the non-optimized screen-printing process, which applies forces on the wafer surface and requires an Ag curing step at 170 °C for a total of 40 min in ambient conditions (see Section S1.1, Supporting Information), the *V*_oc_s of all SHJ single-junction and tandem solar cells are expected to be improved if a thermally evaporated Ag rear contact is used.^67^

Finally, beyond light management techniques suited to standard test conditions, with the view of evaluating the energy output under real-world conditions, energy yield analysis is important for developing tandem solar cells as it can guide other optimization strategies (e.g., perovskite bandgap, bi-faciality).^14,68,69^

## 3 Conclusions

In this study, we designed and developed perovskite/SHJ tandem solar cells, guided by advanced optical simulations, and used (*n*)a-Si:H, (*n*)nc-Si:H, and (*n*)nc-SiO_*x*_:H layers to develop monolithic devices. Different (*i*)a-Si:H passivation strategies were first developed for a (100)-oriented flat c-Si surface and combined with various (*n*)-type layers. Interestingly, bilayers with HPT that exhibit the best (*i*)a-Si:H passivation quality showed degraded minority carrier lifetime when implemented with high hydrogen-diluted nc-Si:H-based layers. Instead, the bilayer without the HPT approach combined with (*n*)nc-Si:H in a symmetrical sample exhibited a high minority carrier lifetime of 16.9 ms.

Mixed-halide WBG active layers were used to develop stable, single-junction perovskite solar cells. Using thin self-assembled hole-transporting monolayers and by passivating the interface between the perovskite and C_60_ layers with choline chloride, solar cells with minimized non-radiative recombination and therefore high *V*_oc_ were prepared. Semi-transparent devices developed using ALD-based SnO_*x*_ and transparent ITO electrodes were integrated with SHJ bottom-cells through an ALD-based NiO_*x*_ interfacial layer to yield 1 cm^2^ monolithic tandems.

Based on optical simulations, anti-reflective coatings and interfacial layers were optimized to minimize optical losses in both sub-cells in order to increase the *J*_sc_ and decrease the current mismatch. As a result, devices with efficiencies above 23% (a maximum of 24.6%) were achieved with all three types of (*n*)-layers with good current matching. Lastly, we explored via optical simulations the potential of various (*n*)-layers with different thicknesses in tandem devices. The use of around 95 nm-thick (*n*)nc-Si:H and 115 nm-thick (*n*)nc-SiO_*x*_:H enables reflection reductions of 1.35 and 1.51 mA/cm^2^, respectively, as compared to 5 nm (*n*)a-Si:H at the intermediate interfaces between the perovskite and c-Si bottom-cells, thus allowing better light coupling into the bottom c-Si solar cells. This marks both (*n*)nc-Si:H and (*n*)nc-SiO_*x*_:H as promising candidates for achieving high-efficiency tandem solar cells. The reduced reflection loss is achieved by minimizing reflection at the intermediate interfaces between the perovskite and SHJ sub-cells through the optimization of interference effects. This technique can be adapted to different tandem designs to realize optimal light management in tandem devices.

## Supporting Information Available

Experimental section; effective lifetime (*τ*_eff_) of symmetrical samples with different (*i*)a-Si:H passivation approaches with (*p*)-layers; *τ*_eff_, i*V*_oc_, and iFF of solar cells before metallization and *V*_oc_ and FF of completed solar cells; EQE and 1 – reflectance (*R*) spectra of single-junction single-side-textured (front-side-flat and rear-side-textured) SHJ solar cells with various front (*n*)-layers; *J*–*V* parameters of single-junction single-side-textured SHJ solar cells with various thicknesses of front (*n*)nc-SiO_*x*_:H layers; UV–vis–NIR and photoluminescence spectra of Cs_0.05_(FA_1–*x*_MA_*x*_)_0.95_Pb(I_1–*x*_Br_*x*_)_3_ perovskite thin films; *J*–*V* curves of perovskite solar cells; PV performance parameters of K_*y*_Cs_0.05_(FA_0.75_MA_0.25_)_0.95–*y*_Pb(I_0.75_Br_0.25_)_3_ perovskite solar cells; *J*–*V* curves of perovskite solar cells with potassium addition; photoluminescence spectra of K_0.05_Cs_0.05_(FA_0.75_MA_0.25_)_0.90_Pb(I_0.75_Br_0.25_)_3_ deposited on the glass substrate upon irradiation with 405 nm; photoluminescence transient of a K_0.05_Cs_0.05_(FA_0.75_MA_0.25_)_0.90_Pb(I_0.75_Br_0.25_)_3_ thin film; energy level diagram of the perovskite/HTL interface; *J*–*V* curves of solar cells using different sALD-processed SnO_*x*_ layer thicknesses; *J*–*V* curves of opaque solar cells using different tALD-processed SnO_*x*_ layer thicknesses; XPS spectra and SEM images of perovskite films without (unpassivated) and with choline chloride surface treatment; absolute photoluminescence spectra of perovskite thin films; PV parameters of perovskite solar cells using choline chloride interfacial passivation from precursor solutions of different concentrations; EQE spectra; *J*–*V* curves of opaque (Al) and semi-transparent (ITO) solar cells; image of a fabricated tandem solar cell and cross-sectional SEM images of perovskite top and silicon-bottom cells; EFM measurement; bright field transmission electron microscopy images of monolithic tandem devices; EQE spectra; refractive indices of (*n*)-type layers and perovskite and c-Si active layers; comparisons between the measured EQE and the simulated absorptance of tandem solar cells with different (*n*)-layers; light management in fully-textured monolithic tandem solar cells and image of SHJ wafer diced for top-cell fabrication to prepare tandem solar cells (PDF)
