# Supplementary material for: Optical Simulation-Aided Design and Engineering of Monolithic Perovskite/Silicon Tandem Solar Cells
Source: ACS Appl Energy Mater. 2023 May 3;6(10):5217–29. doi: 10.1021/acsaem.3c00136 (PMC10206623; doi:10.1021/acsaem.3c00136)
Supplement: Supplementary file 1 — ae3c00136_si_001.pdf [file ae3c00136_si_001.pdf]

Supporting Information

Optical Simulation-Aided Design and Engineering of Monolithic Perovskite/Silicon Tandem Solar Cells

Yifeng Zhao,[1] Kunal Datta,[2] Nga Phung,[3] Andrea E. A. Bracesco,[3] Valerio Zardetto,[4] Giulia Paggiaro,[1] Hanchen Liu,[1] Mohua Fardousi,[1] Rudi Santbergen,[1] Paul Procel Moya,[1] Can Han,[1] Guangtao Yang,[1] Junke Wang,[2] Dong Zhang,[2,4] Bas T. van Gorkom,[2] Tom P. A. van der Pol,[2] Michael Verhage,[2] Martijn M. Wienk,[2] Wilhelmus M. M. Kessels,[3] Arthur Weeber,[1,5] Miro Zeman,[1] Luana Mazzarella,[1] Mariadriana Creatore,[3,6] René A. J. Janssen,[2,7] Olindo Isabella[1*]

[1] Photovoltaic Materials and Devices group, Delft University of Technology, partner in Solliance, 2628 CD Delft, The Netherlands

[2] Molecular Materials and Nanosystems, Institute for Complex Molecular Systems, Eindhoven University of Technology, partner in Solliance, P.O. Box 513, 5600 MB Eindhoven, The Netherlands

[3] Department of Applied Physics and Science of Education, Eindhoven University of Technology, partner in Solliance, Eindhoven, P.O. Box 513, 5600 MB The Netherlands

[4] TNO, partner in Solliance, 5656 AE Eindhoven, The Netherlands

[5] TNO Energy Transition – Solar Energy, P.O. Box 15, 1755 ZG Petten, The Netherlands

[6] Eindhoven Institute for Renewable Energy Systems, P.O. Box 513, 5600 MB Eindhoven, The Netherlands

[7] Dutch Institute for Fundamental Energy Research, 5612 AJ Eindhoven, The Netherlands

(corresponding author: Olindo Isabella. E-mail: O.Isabella@tudelft.nl)

1. EXPERIMENTAL SECTION

1.1 Sample preparation

The silicon wafers used for the passivation optimizations and solar cells are Topsil (*n*)-type double-side-polished FZ <100> c-Si wafers with a range of resistivity (3 ± 2 Ω·cm) and thickness (280 ± 20 μm). Before loading wafers into the PECVD cluster tool, their surface was cleaned by sequentially dipping the wafers into 99% room-temperature nitric acid (HNO_3), 69.5% 110 °C HNO_3, and then 0.55% hydrofluoric acid (HF). Both (*i*)a-Si:H and doped Si thin-film layers were deposited via multi-chamber radio-frequency (RF, 13.56 MHz) or very-high-frequency (VHF, 40.68 MHz) PECVD cluster tools (Elettrorava S.p.a.). The deposition parameters and thicknesses/duration for optimized (*i*)a-Si:H layers and hydrogen-plasma treatment (HPT) can be found in Table S1. For (*i*)a-Si:H layers, two deposition methods, *i.e.* without H_2 added (*i*-1) and with H_2 added (*i*-2) to the silane, were used. The detailed parameters for doped layers can be found in our previous studies.[1,2] A geometrical factor of 1.7 was used for calculating the deposition durations for the textured surface. For passivation optimizations, layers were symmetrically deposited on the substrate.

Table S1. PECVD deposition parameters of optimized (*i*)a-Si:H layers and the HPT.

	i -1	*i*-2	HPT
$f[H_2]/f[SiH_4]$ (sccm/sccm)	0/40	30/10	200/0
Pressure (mbar)	0.7	1.4	2.2
Power density (mW/cm^2)	20.8	20.8	41.7
Temperature (°C)	160	160	160
Thickness (nm)	10/5*	5	duration 20 s

* 10 nm and 5 nm (*i*)a-Si:H-1 are used for mono- and bi-layer passivation strategies, respectively.

To prepare the single-side-textured substrates for solar cell fabrications, we deposited PECVD (Novellus Systems Inc.) grown SiN_x on one side of polished c-Si wafers. SiN_x is comparatively etching-resilient to diluted tetramethylammonium hydroxide (TMAH) solution. After the texturing of the bare c-Si side that is not covered with SiN_x, wafers were dipped into a buffered-HF (BHF) solution to completely remove the SiN_x. This produced single-side-textured wafers that feature a front side (100)-oriented flat surface and a rear side (111)-oriented textured surface. Before the

deposition of Si thin-film layers, the same wafer cleaning procedure mentioned above was performed. We first deposited the *i*/*n* stack and then the *i*/*p* stack to form solar cell precursors. Subsequently, RF-magnetron sputtered (Polyteknic AS) tin-doped indium oxide (ITO, 90 wt% In_2O_3 and 10 wt% SnO_2) layers were deposited on the front and rear sides of the single-junction cell precursors with thicknesses of 75 nm and 150 nm, respectively. Lastly, solar cells were finished with screen-printed Ag contacts and cured in an oven under an air atmosphere at 170 °C for 40 minutes.

To prepare the perovskite single junction solar cell and top cell in tandems, all materials were used as received without purification and stored in an inert environment unless stated otherwise. 2PACz (> 98%) self-assembled monolayer, PbI_2 (> 99.99%), and $PbBr_2$ (> 98%) were purchased from TCI. FAI (> 99.99%) and MABr (> 99.99%) were purchased from Greatcell Solar Materials. KI (ultra-dry, 99.998%) was purchased from Alfa Aesar. CsI (beads, 99.999%), choline chloride (> 99%), DMF (99.8%), DMSO (99.9%), 2-propanol and anisole (99.7%) were purchased from Sigma Aldrich. C_{60} (99.95%) was purchased from SES Research and bathocuproine (BCP, 99%) was purchased from Lumtec.

2PACz was dissolved in anhydrous ethanol at a concentration of 0.3 mg/mL by sonication prior to use. To prepare the perovskite precursor solution, PbI_2 (691.5 mg/mL) and $PbBr_2$ (550.5 mg/mL) were each dissolved overnight at 60 °C in solvent mixtures containing DMF and DMSO in a volumetric ratio of 4:1. Stock solutions of CsI (389.7 mg/mL) in DMSO and KI (249.0 mg/mL) in DMF:DMSO (4:1 v/v) were prepared and stirred overnight at 60 °C. The solutions were cooled to room temperature, following which 936 μL PbI_2 was added to 199.9 mg FAI and 936 μL $PbBr_2$ was added to 133.1 mg MABr. The resulting solutions ($FAPbI_3$ and $MAPbBr_3$) were stirred at 60 °C for approx. 1 h and then cooled to room temperature. For 21%/25% Br-containing perovskite, 752 μL/714 μL $FAPbI_3$ and 200 μL/238 μL $MAPbBr_3$ were mixed with 48 μL CsI and 48 μL KI; the resulting solution was stirred at 60 °C for approx. 1 h and cooled to room temperature prior to use. Choline chloride was dissolved in 2-propanol (1 mg/mL) and stirred overnight at 60 °C.

Single-junction perovskite solar cells were prepared on glass/ITO substrates (Naranjo Substrates 15 – 17 Ω/sq.). The substrates were first cleaned in an ultrasonic bath of acetone, followed by scrubbing with sodium dodecyl sulphate (Acros, 99%) soap solution in deionized water, sonication in the soap solution, rinsing in deionized water, followed by sonication in 2-

propanol. Prior to use, the substrates were exposed to UV-ozone treatment for 30 min after which they were transferred to a N_2-filled glove-box. 2PACz hole transport layer was spin-coated at 3000 rpm for 30 s followed by thermal annealing at 100 °C for 10 minutes. The samples were allowed to cool down for 5 –10 min following which the perovskite film was deposited by spin-coating 150 µL of the precursor at 4000 rpm (5 s to ramp to 4000 rpm) for 35 s. At approx. 25 s from the beginning of the spin-coating, 300 µL of anisole was cast onto the spinning substrate. The substrates were immediately annealed at 100 °C for 30 min. Then, 20 nm C_{60} and 8 nm BCP were sequentially evaporated at a rate of 0.5 Å/s following which a 100 nm Ag electrode was thermally evaporated. Where mentioned, atomic layer deposited (ALD) SnO_x (described below) was used to replace BCP.

To develop the tandem devices, the wafers were diced into 2.5 cm × 2.5 cm sized samples (Figure S27) prior to deposition of the perovskite top cell. Where mentioned, approx. 8-nm-thick NiO_x was deposited using atomic layer deposition on the ITO layer (30 nm). The deposition was done at a base pressure of 5 × 10^{-6} mbar in a home-built reactor using nickel bis(*N*,*N*'-di-tert-butylacetamidinate) ($Ni(tBu\text{-}MeAMD)_2$) as the nickel source and water as the co-reactant. The precursor bubbler was maintained at 90 °C and Ar flow was used for bubbling. The substrate temperature approached 150 °C during the deposition. 2PACz, perovskite, choline chloride, and C_{60} layers were deposited as described before. Following that, either spatial atomic layer deposition (sALD) or temporal ALD (tALD) were used to deposit SnO_x buffer layer of variable thickness. Tetrakis(dimethylamino)tin(IV) was used as the tin source and water as the co-reactant. In case of sALD, a nitrogen curtain was used to isolate the two half-reaction steps. A 180-nm-thick ITO layer was deposited using RF sputtering process at a rate of 0.3 Å/s. Finally, a 100-nm-thick Ag perimeter contact and a 120 nm-thick MgF_2 anti-reflective coating were thermally evaporated to complete the tandem device.

1.2 Characterizations

Spectroscopic Ellipsometry (SE) (M-2000DI system, J.A. Woollam Co., Inc.) was used to check the thicknesses of deposited layers on flat wafers and to extract the refractive index (*n*) and the optical bandgap (E_{04}) that is the energy at which the optical absorption coefficient reaches 10^4 cm^{-1}. The passivation quality of symmetrical samples and solar cell precursors was measured with Sinton WCT-120 under either quasi-steady-state photoconductance (QSSPC) mode or transient

photoconductance decay (Transient PCD) mode. To ensure good passivation quality comparability among different samples,[3] the thicknesses of symmetrically deposited (*i*)a-Si:H layers were kept constant at around 10 nm. To evaluate the microstructure quantitative composition and the hydrogen bonding configurations of the deposited (*i*)a-Si:H films, Fourier-transform infrared (FTIR) spectroscopy was used. For FITR characterizations, around 30-nm-thick (*i*)a-Si:H layers were deposited on flat <100> (*n*)-type c-Si Czochralski (CZ) wafers with a thickness of 525 ± 20 µm and resistivity of 15 ± 5 Ω·cm. From FTIR measurements, we extracted the microstructure factor (R_{SM}) that is defined for Si-H stretching modes (SM) of (*i*)a-Si:H layers as:[4]

$$R_{\mathrm{SM}} = I_{\mathrm{HSM}}/(I_{\mathrm{LSM}} + I_{\mathrm{HSM}}) \quad (1)$$

where LSM and HSM stand for low-frequency SM and high-frequency SM, respectively, and I is the integrated absorbance:[5]

$$I = \int \left(\frac{\alpha}{\omega}\right) d\,\omega \quad (2)$$

α is the absorption coefficient. Further, we calculated the hydrogen content (C_H) of the film as the following:

$$C_{\mathrm{H}} = \frac{N_{\mathrm{H}}}{N_{\mathrm{Si}}} = \frac{I_{\mathrm{LSM}} \cdot A_{\mathrm{LSM}} + I_{\mathrm{HSM}} \cdot A_{\mathrm{HSM}}}{N_{\mathrm{Si}}} \quad (3)$$

where N_H and N_{Si} (5×10^{22} cm^{-3}) are the density of H and Si atoms, respectively. A_{LSM} and A_{HSM} are proportionality constants and $A_{LSM} = 9.0 \times 10^{19}$ cm^{-2} and $A_{HSM} = 2.2 \times 10^{20}$ cm^{-2}.[5]

UV-vis-NIR spectra to characterize the absorption behavior of perovskite thin films were measured using PerkinElmer Lambda 1050 UV-vis-NIR spectrophotometer. Scanning electron microscopy images were acquired using FEI Quanta 3D FEG microscope, operated with a 5 kV electron beam and a secondary electron detector. Steady-state photoluminescence spectra were acquired using a xenon lamp monochromatized at 600 nm to excite the perovskite film deposited on glass substrates. The spectrum was measured using an Edinburgh FLSP920 double-monochromator luminescence spectrometer. Time-resolved photoluminescence spectroscopy was conducted using an Edinburgh Instruments LifeSpec-PS spectrophotometer with an excitation wavelength of 400 nm using a pulsed diode laser (LDH0C driven by PDL-800). Photoluminescence

measurements during ageing were conducted using a custom-built setup. The films were mounted into a sealed chamber to maintain inert atmosphere. Blue LED illumination (Thorlabls M405L4, 405 nm, driven by Thorlabs DC4104) with intensity calibrated using a silicon photodiode was used to irradiate the film and also as an excitation source for photoluminescence measurements. The emission, filtered by a 645 nm long-pass filter, was focused onto an optical fiber connected to a spectrometer (Avantes Avaspec-2048×14) operated on a custom-built code in the LabVIEW environment. Sensitive photocurrent spectroscopy to observe sub-bandgap states was done using an Osram 64655 HLX 250 W halogen lamp as an illumination source. The light was chopped using an Oriel 3502 chopper and was subsequently passed through a monochromator (Oriel, Cornerstone 260) and appropriate sorting filters. The solar cell response was recorded as a voltage from a pre-amplifier (Stanford Research, SR570) using a lock-in amplifier (Stanford Research, SR830). The measurements were calibrated using Si and InGaAs reference cells. To illuminate the solar cells and observe light-induced instability, a 532 nm CW laser (Thorlabs, CPS532-C2, driven by LDS 5) was used with an intensity and illumination area matched to 1-Sun equivalent intensity and the solar cell area using a set of neutral density filters and fisheye lenses. The data was normalized to the drop in photocurrent that marks the band-edge in pristine solar cells. After laser illumination, the spectra were scaled for the signal produced from pristine solar cells in order to estimate the change in the above-gap EQE. Conductive atomic force microscopy measurements were conducted with a Veeco Dimension MultiMode microscope connected to a Nanoscope III controller in tapping mode using PPP-NCH probes. The probes were sputtered with 3 nm tantalum seed layer and 30 nm platinum. A TUNA head was used to apply bias and record currents from the layers with contact mode configuration at a scan rate of 0.5 Hz. X-ray photoelectron spectroscopy was conducted using Thermo Scientific K-Alpha with 180° double-focusing hemispherical analyzer and a 128-channel detector. Monochromatic Al Kα (1486.6 eV) radiation was used and the X-ray spot was 400 µm. For the surface analysis, a survey scan was measured for 15 scans with a pass energy of 200 eV. High resolution scans (30 scans) for specific elements were conducted with a pass energy of 50 eV. Absolute photoluminescence spectroscopy was done using 455 nm LED (Thorlabs, M455F3-455 fiber coupled LED) source to excite the perovskite film through an optical fiber placed in an integrating sphere (Avantes, AvaSphere-30-REFL) fitted with a 550 nm short-pass filter (Edmund Optics). The excitation intensity was calibrated to 1 Sun equivalent intensity. The spectrum is

measured through an optical fiber connected to a calibrated AvaSpec-HERO spectrometer (Avantes, AVASPEC- HSC1024X58TEC-EVO) using a 550 nm long-pass filter.

Current density versus voltage measurements were done using a tungsten-halogen lamp filtered by a UV filter (Schott GG385) and a daylight filter (Hoya LB120) with the intensity adjusted to 100 mW/cm^2. A shadow mask with an aperture smaller than the active area (0.0676 cm^2 for single-junction perovskite solar cells and 1 cm^2 for monolithic tandem solar cells) was used to define the illumination area of the cell. The solar cells were operated between + 1.5 V (+ 2.0 V for monolithic tandem solar cells) and – 0.5 V at a scan rate of 0.25 V/s by a Keithley 2400 source meter. For external quantum efficiency measurements, a modulated monochromatic probe light (Philips focusline 50 W) was used to illuminate the solar cells through an aperture (1 mm radius). The response was recorded and converted to EQE using a calibrated silicon reference cell. Light- and voltage-biasing was used to isolate the EQE of the individual sub-cells; a 530 nm (perovskite) or 940 (silicon) bias light and a forward voltage bias close to the V_{oc} of the single-junction cell was used. The silicon single-junction solar cell was measured using a AAA class WACOM WXS-90S-L2 solar simulator under standard-test-conditions. A shadow mask with an aperture area 4.05 cm^2 was used.

1.3 Advanced optical simulations

Genpro4,[6] which combines ray-tracing and wave-optics, was used for optical simulations of the perovskite/SHJ tandem solar cells. Spectroscopic ellipsometry was used to extract optical constants for the layers used in this study. The data for Ag and c-Si are taken from literature reports.[7,8]

2. ADDITIONAL FIGURES AND TABLES

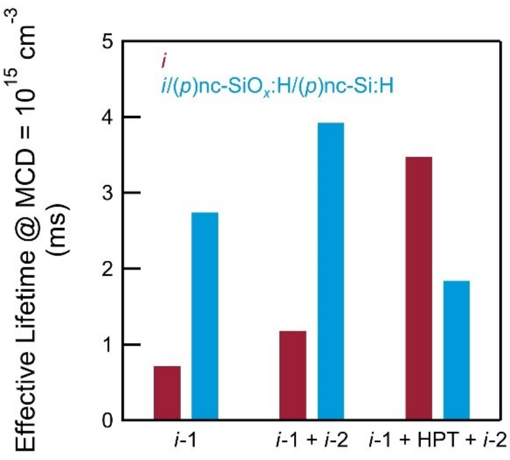

Figure S1. The effective lifetime (τ_{eff}) of symmetrical samples with different (*i*)a-Si:H passivation approaches with (*p*)-layers.

Table S2. The τ_{eff}, iV_{oc} and iFF of solar cells before metallization and V_{oc} and FF of completed solar cells.

Cell types	With ITO on both sides (before metallization)			Completed solar cells	
	τ_{eff} @ MCD = 10^{15} cm^{-3} (ms)	iV_{oc} (mV)	iFF (%)	V_{oc} (mV)	FF (%)
(*n*)a-Si:H (5 nm)	2.06	710	82.8	704	80.7
(*n*)nc-Si:H (20 nm)	7.22	722	85.2	714	80.7
(*n*)nc-SiO$_x$:H (40 nm)	1.31	716	79.4	694	77.5

This table indicates drops from iV_{oc} to V_{oc} and iFF to FF when solar cells are finalized with screen-printed Ag contacts. We attribute these drops, at least in part, to our lab-standard non-optimized screen printing process [9], which degrades the passivation quality. Additionally, the observed drops may be also due to insufficient selectivity of electron and/or hole contact stacks used in solar cells [10].

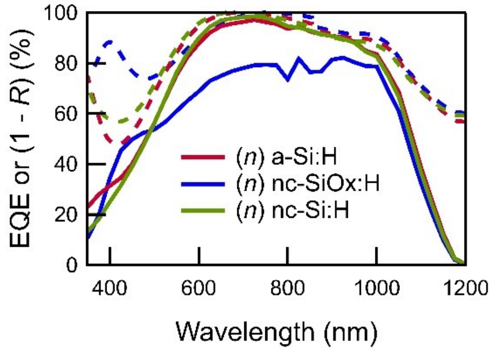

Figure S2. EQE and 1 – reflectance (*R*) spectra of single-junction single-side-textured (front-side-flat and rear-side-textured) SHJ solar cells with various front (*n*)-layers. Solid lines represent EQE

and dashed lines represent $(1 - R)$. Note, for the cell with (n)nc-SiO_x:H, the overall low EQE in the range from around 500 nm to 1000 nm is ascribed to significant recombination.

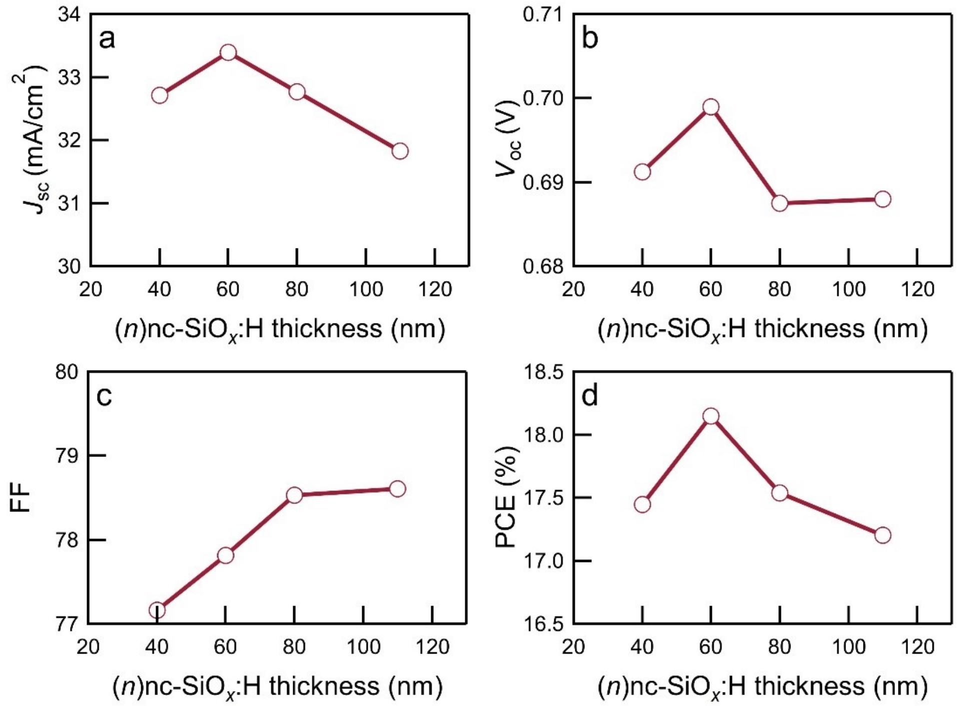

Figure S3. The *J-V* parameters of single-junction single-side-textured SHJ solar cells with various thicknesses of front (n)nc-SiO_x:H layers. The results represent averaged parameters of three to five solar cells.

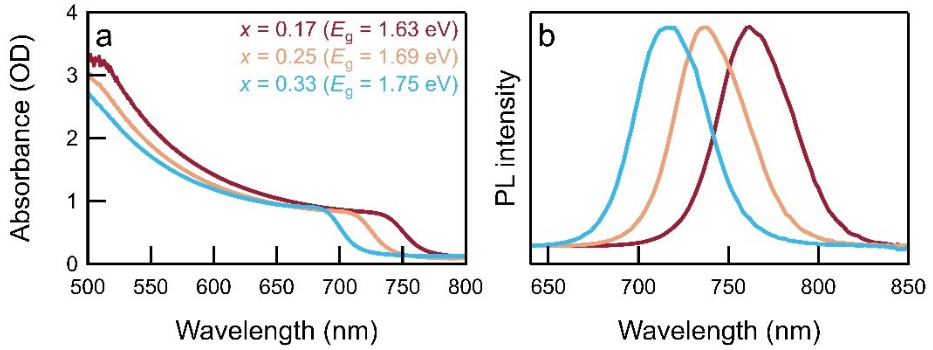

Figure S4. (a) UV-vis-NIR and (b) photoluminescence spectra of $Cs_{0.05}(FA_{1-x}MA_x)_{0.95}Pb(I_{1-x}Br_x)_3$ perovskite thin films.

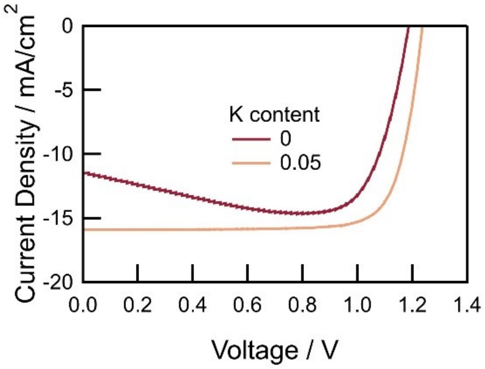

Figure S5. *J–V* curves of perovskite solar cells ($K_yCs_{0.05}(FA_{0.55}MA_{0.45})_{0.95-y}Pb(I_{0.55}Br_{0.45})_3$).

Table S3. PV performance parameters of $K_yCs_{0.05}(FA_{0.75}MA_{0.25})_{0.95-y}Pb(I_{0.75}Br_{0.25})_3$ perovskite solar cells.

y	V_{oc} (V)	J_{sc} (mA/cm^2)	FF -	PCE (%)
0	1.16	19.0	0.81	17.9
0.05	1.18	19.0	0.81	18.2
0.10	1.17	19.2	0.79	17.7

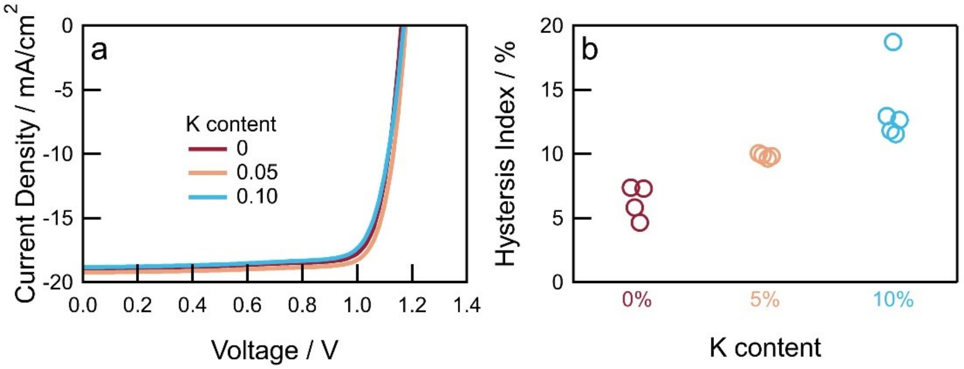

Figure S6. (a) Current density versus voltage curves of perovskite solar cells ($K_yCs_{0.05}(FA_{0.75}MA_{0.25})_{0.95-y}Pb(I_{0.75}Br_{0.25})_3$) with potassium addition. (b) Hysteresis index of solar cells calculated as the absolute percentage difference between forward and reverse scans (($PCE_{reverse} - PCE_{forward})/PCE_{reverse}$).

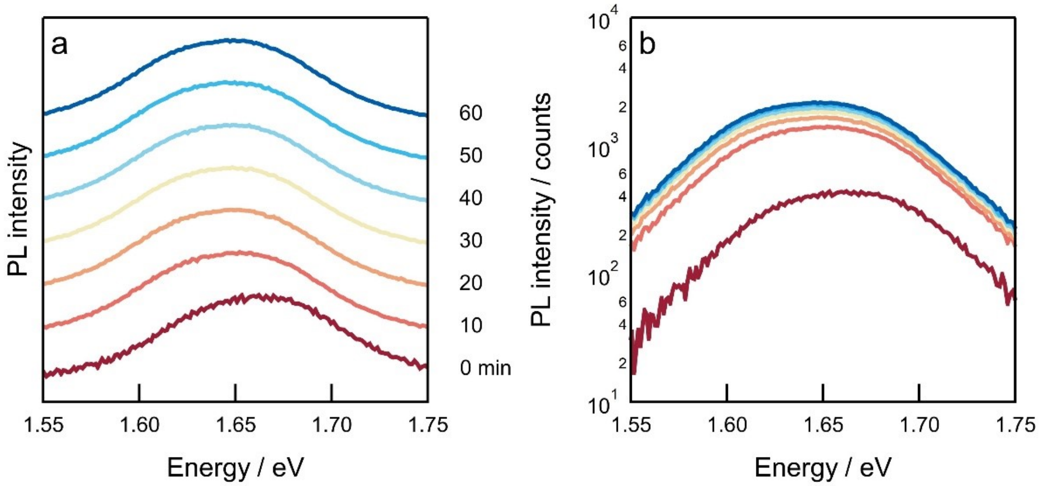

Figure S7. Photoluminescence spectra of $K_{0.05}Cs_{0.05}(FA_{0.75}MA_{0.25})_{0.90}Pb(I_{0.75}Br_{0.25})_3$ deposited on glass substrate upon irradiation with 405 nm (~ 4 Sun equivalent intensity). (a) Normalized spectra at different periods of illumination. Spectra are plotted on a linear y-axis and offset vertically. (b) Spectra shown in panel (a) without normalization plotted on a logarithmic y-axis.

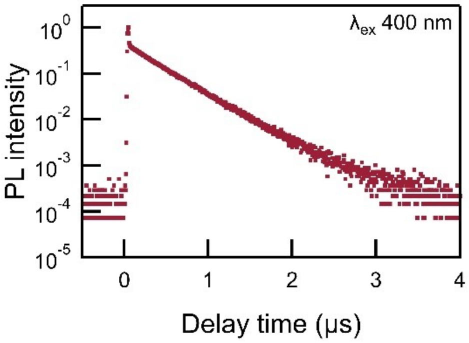

Figure S8. Photoluminescence transient of a $K_{0.05}Cs_{0.05}(FA_{0.75}MA_{0.25})_{0.90}Pb(I_{0.75}Br_{0.25})_3$ thin film.

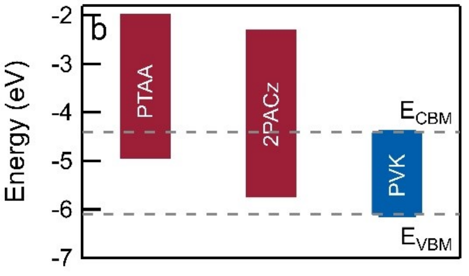

Figure S9. Energy level diagram of perovskite/HTL interface.

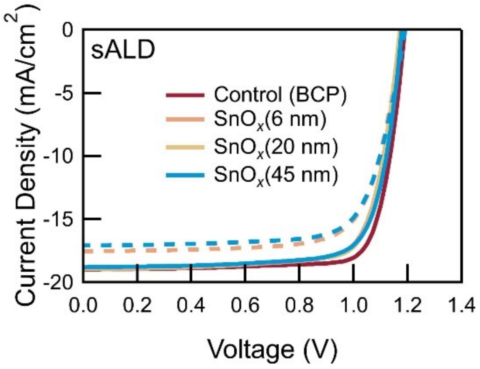

Figure S10. *J*-*V* curves of solar cells using different sALD-processed SnO_x layer thicknesses. Solid lines represent opaque solar cells and dashed lines represent semi-transparent solar cells illuminated from the hole-transport layer interface.

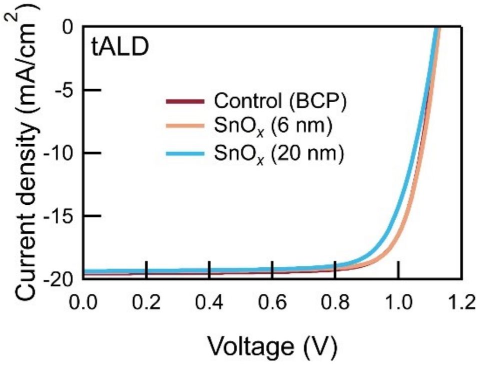

Figure S11. *J*-*V* curves of opaque solar cells using different tALD-processed SnO_x layer thicknesses.

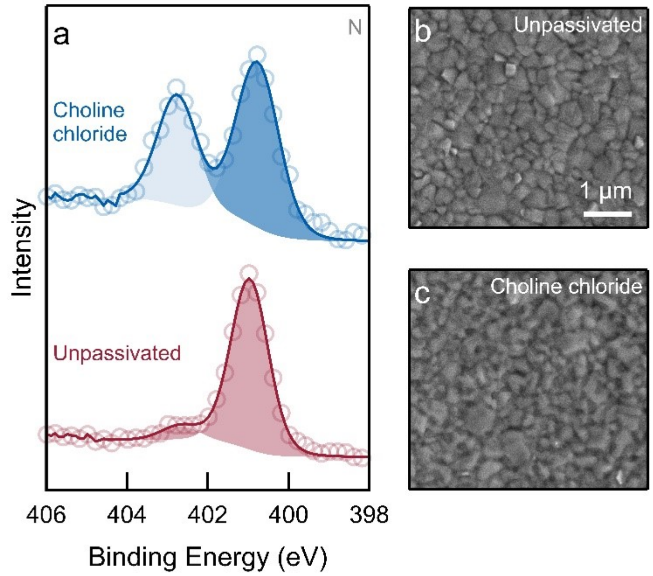

Figure S12. (a) N 1*s* XPS spectra of perovskite film without (unpassivated) and with choline chloride surface treatment. (b-c) SEM images of perovskite film without (unpassivated) and with choline chloride surface treatment.

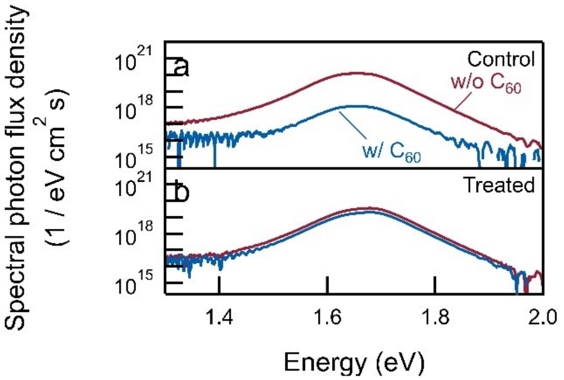

Figure S13. Absolute photoluminescence spectra of perovskite thin films w/o (control, panel a) or w/ (treated, panel b) choline chloride deposition, before (w/o C_{60}) and after (w/ C_{60}) the application of a C_{60} layer on top.

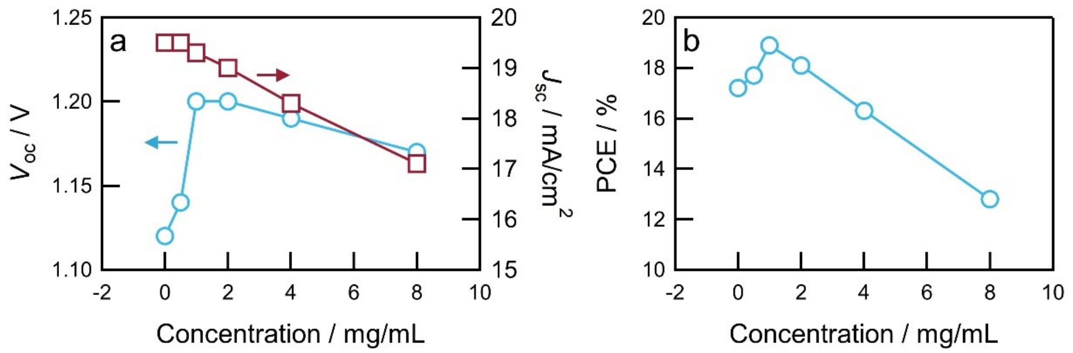

Figure S14. PV parameters of perovskite solar cells using choline chloride interfacial passivation from precursor solutions of different concentrations. (a) Open-circuit voltage and short-circuit current density. (b) Power conversion efficiency.

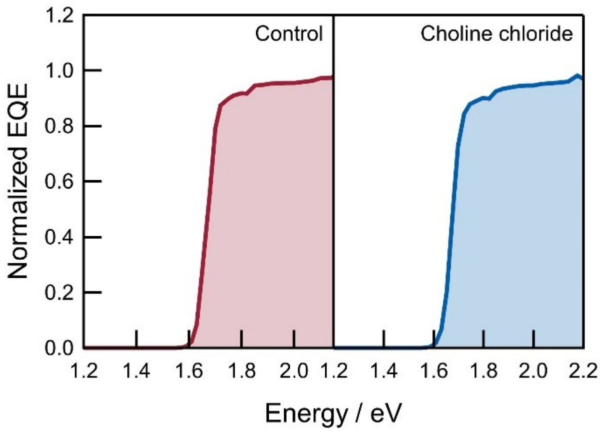

Figure S15. EQE spectra shown in Figure 2e plotted on a linear y-axis. Shaded region corresponds to the EQE spectrum of a pristine solar cell and solid line represents the spectrum of a solar cell stressed with light for 1 h (control) and 16 h (choline chloride).

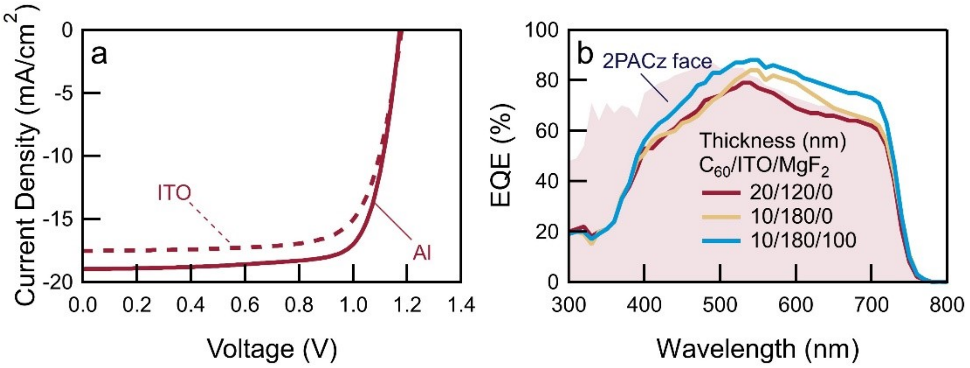

Figure S16. (a) Current-density versus voltage curves of opaque (Al) and semi-transparent (ITO) solar cells. (b) EQE spectra of semi-transparent solar cells illuminated from the 2PACz face

(shaded), and from the C_{60} face (lines) with various thicknesses of C_{60}, ITO, and MgF_2. The other approximate layer thicknesses are: ITO (180 nm), 2PACz (< 5 nm), and perovskite (460 nm).

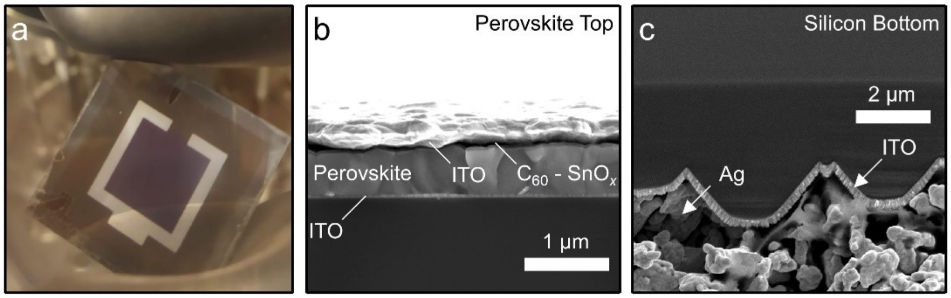

Figure S17. (a) Image of a fabricated tandem solar cell. (b-c) Cross-sectional SEM images of (b) perovskite top- and (c) silicon bottom-cells.

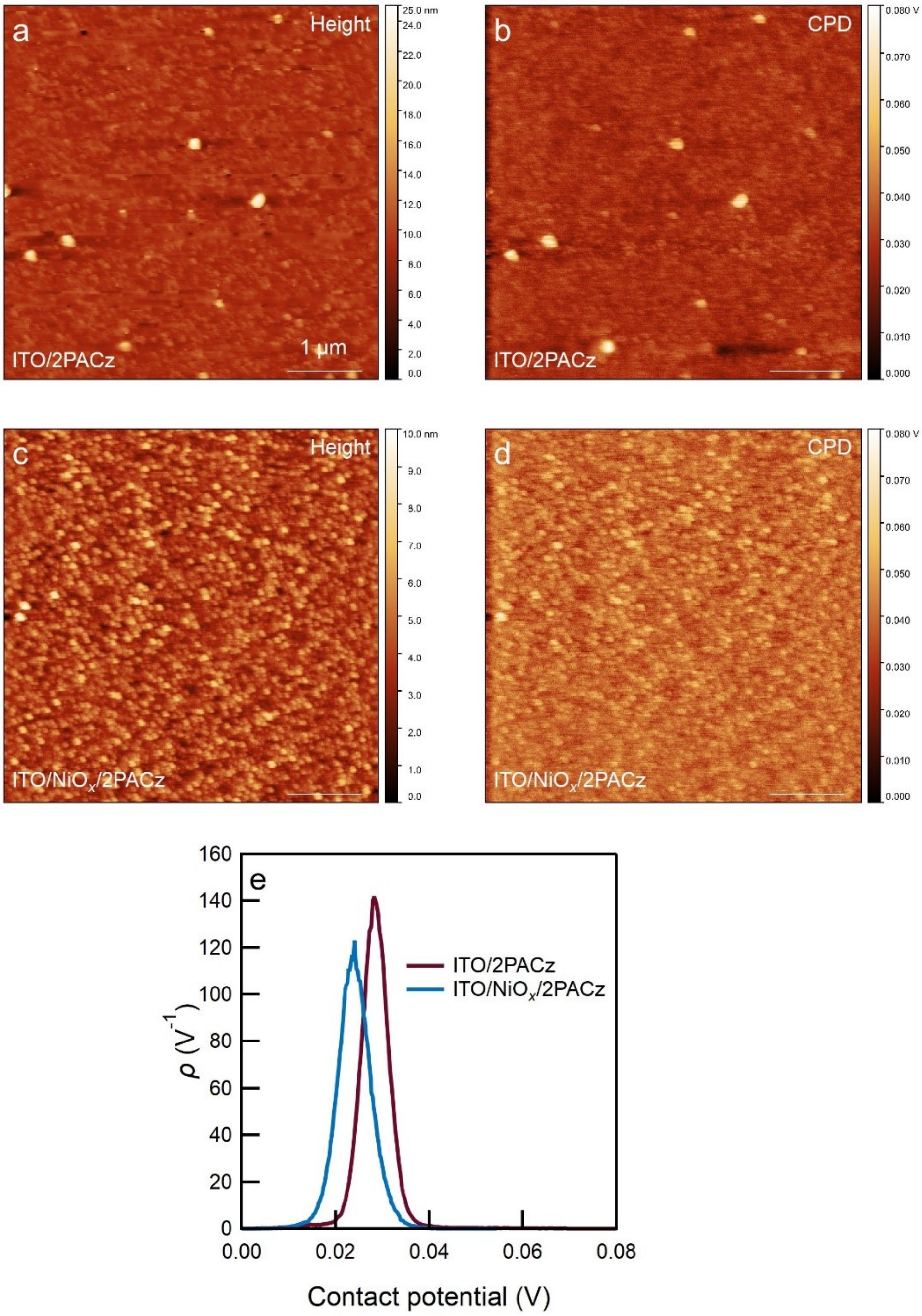

Figure S18. EFM measurement on (a,b) ITO/2PACz and (c,d) ITO/NiO_x/2PACz showing (a,c) height map and (b,d) contact potential difference (CPD) map on the same spot. (e) Statistical distribution of contact potential value for the corresponding maps in (b) and (d).

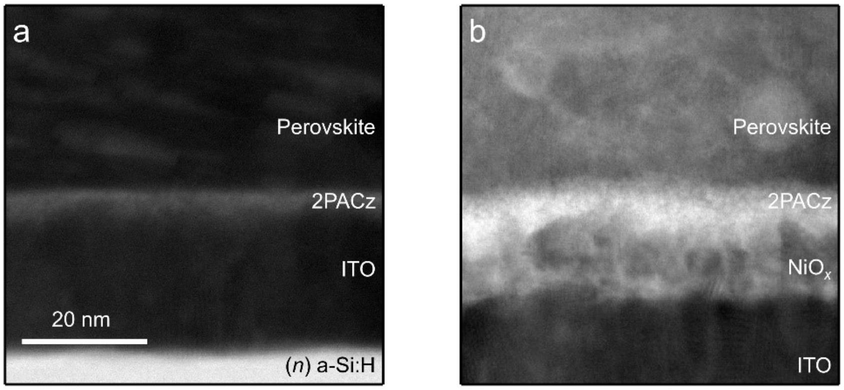

Figure S19. Bright field transmission electron microscopy images of monolithic tandem devices focused on the (a) ITO/2PACz and (b) ITO/NiO_x/2PACz interfaces.

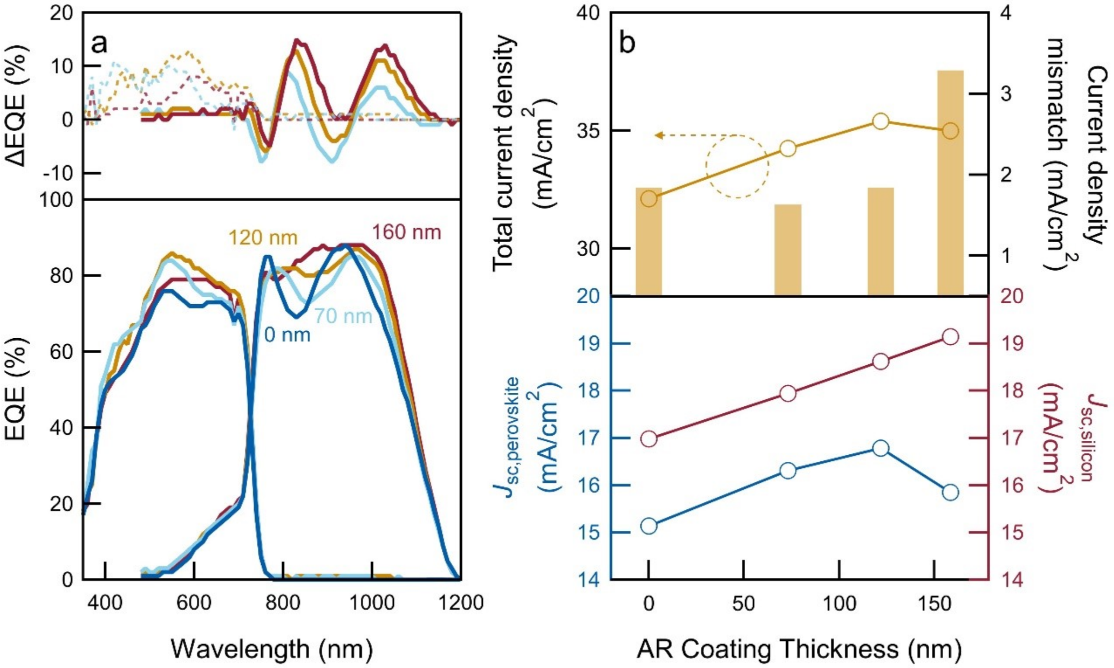

Figure S20. (a) EQE spectra of tandem solar cells using different MgF_2 layer thicknesses. Top sub-figure shows the difference in EQE compared to that of the cell without MgF_2 coating (0 nm). Dashed lines represent the top-cell and solid lines represent the bottom-cell in the top sub-figure. (b) J_{sc} contributions of perovskite and SHJ sub-cells in tandem devices calculated from EQE in panel (a). Top sub-figure shows the total current density ($J_{sc,perovskite} + J_{sc,silicon}$) and current density mismatch ($|J_{sc,perovskite} - J_{sc,silicon}|$). In all cases, the perovskite top-cell is current limiting. It must be

noted that the perovskite bandgap used is 1.69 eV. C_{60} layer is 20 nm thick and SnO_x layer is 45 nm thick.

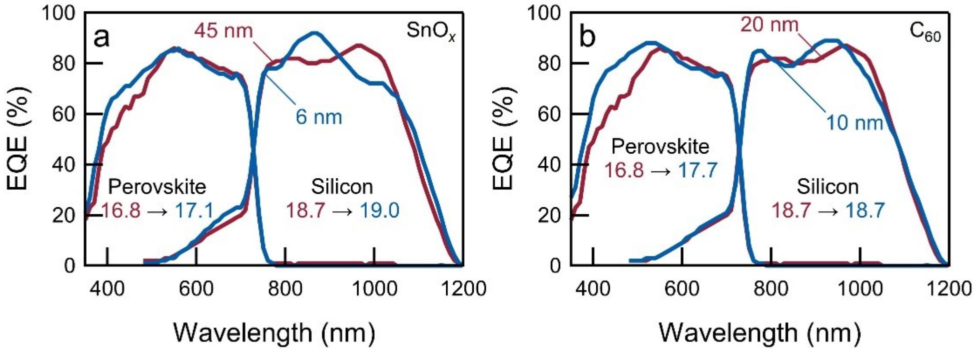

Figure S21. EQE spectra of tandem solar cells using (a) 45 nm or 6 nm SnO_x (20 nm C_{60} and 120 nm MgF_2), or (b) 10 nm or 20 nm C_{60} (45 nm SnO_x and 120 nm MgF_2) layers. Values show the J_{sc} contributions of perovskite or SHJ sub-cells. It must be noted that the perovskite bandgap used is 1.69 eV.

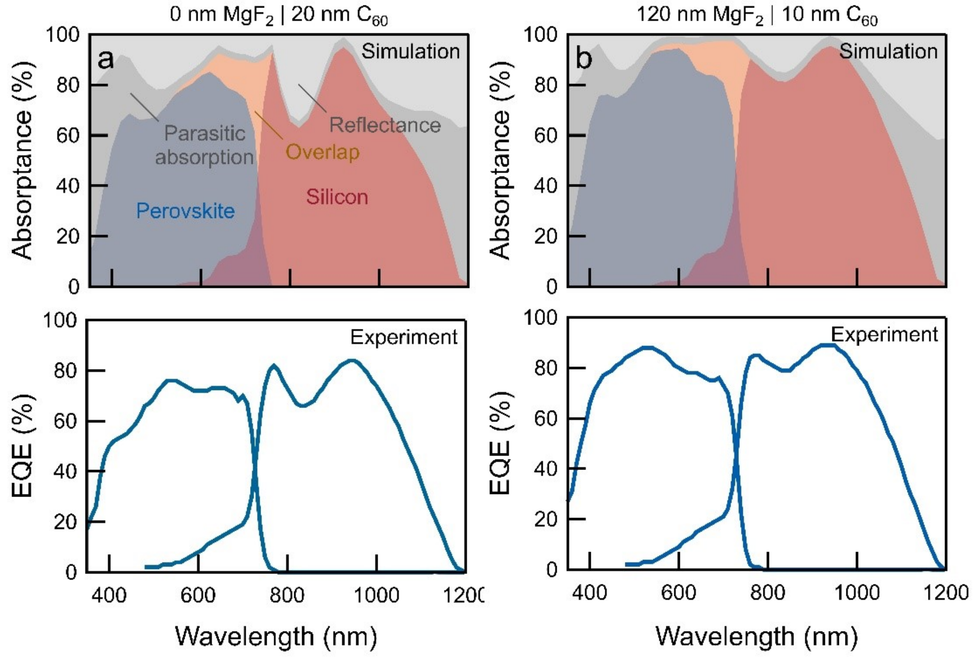

Figure S22. Experimentally determined EQE and simulated absorptance spectra of tandem solar cells using (a) 0 nm MgF_2 and 20 nm C_{60}, or (b) 120 nm MgF_2 and 10 nm C_{60}. It must be noted that the perovskite bandgap used is 1.69 eV. A 45 nm SnO_x layer was used in all cases.

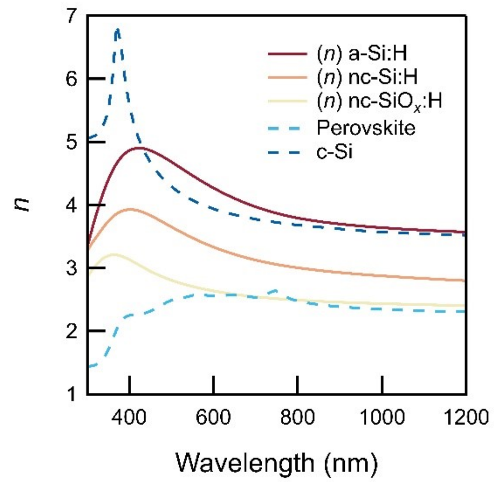

Figure S23. Refractive indices of (*n*)-type layers and perovskite and c-Si active layers. The data of c-Si is taken from literature [7].

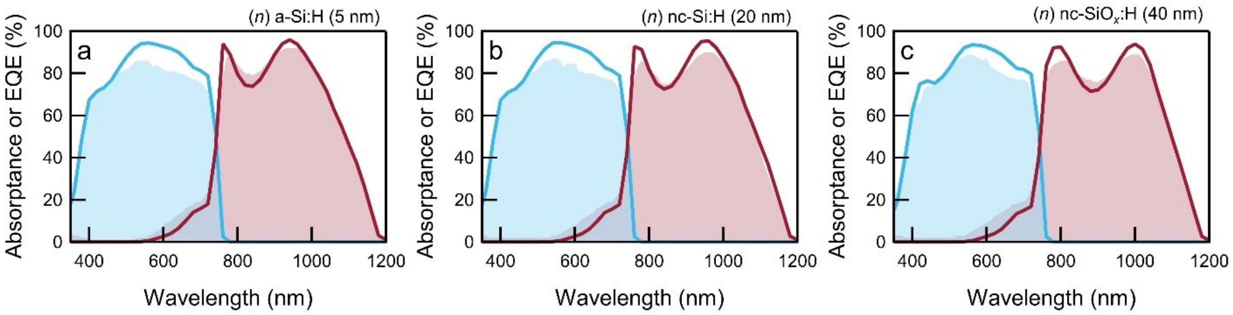

Figure S24. Comparisons between the measured EQE and the simulated absorptance of tandem solar cells with different (*n*)-layers. Shaded areas represent measured EQE values, and the solid lines are simulated absorptance curves. The mismatches between the EQE and the simulated absorptance could be attributed to the following: choline chloride and 2PACz were not considered in the simulations due to difficulty in extracting their optical properties with nearly negligible thicknesses; the overestimated extinction coefficient of perovskite absorber thus the high simulated absorption of perovskite top cell; the non-ideal collection efficiency of charge carriers in real devices while the simulation considers only optical effects.

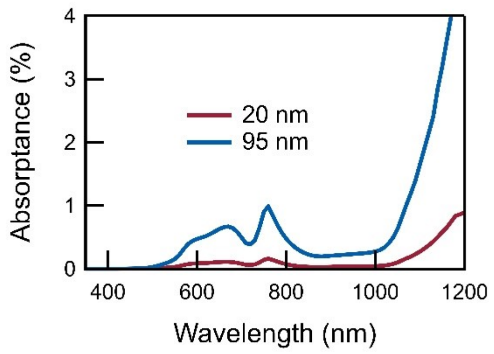

Figure S25. Simulated absorptance profile of (n)nc-Si:H layers of 20 nm or 95 nm thicknesses used in a tandem solar cell.

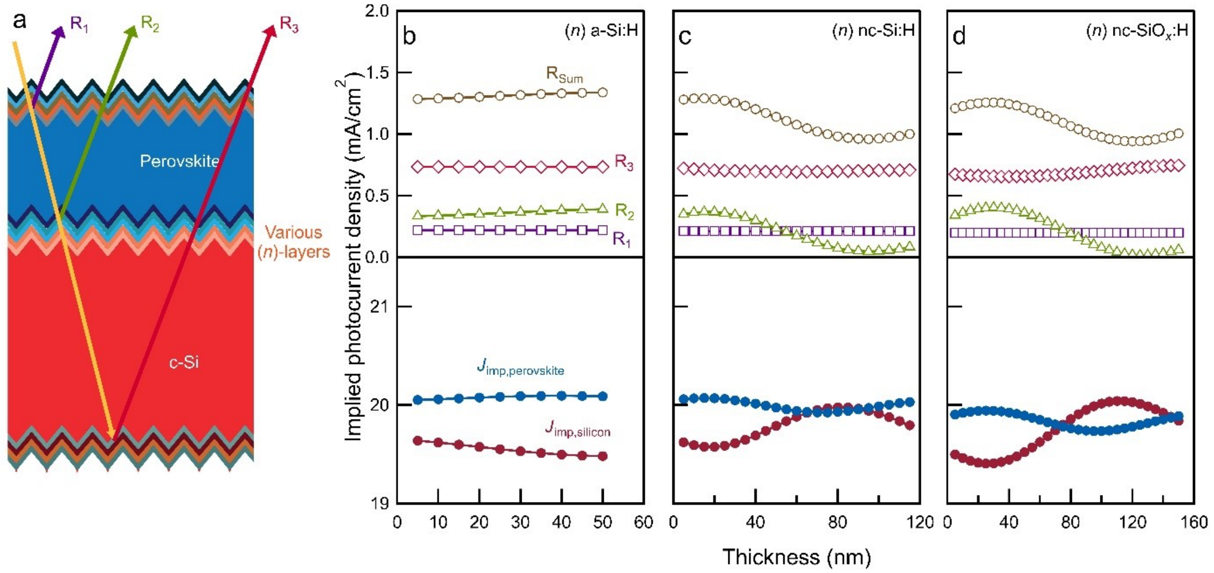

Figure S26. Light management in fully-textured monolithic tandem solar cells. (a) Schematic of the perovskite/SHJ tandem solar cell for optical simulations, and (b – d) implied photocurrent density of the perovskite top-cell, SHJ bottom-cell and reflected light as function of (n)-layer thickness and types of (n)-layer.

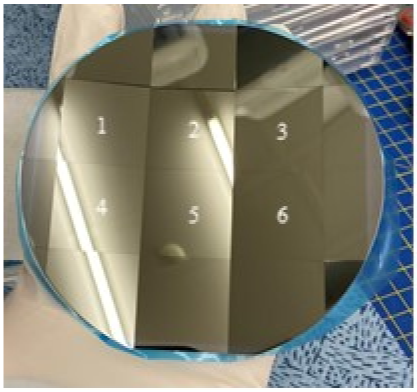

Figure S27. Image of SHJ wafer diced for top-cell fabrication to prepare tandem solar cells.

3. REFERENCES

(1) Zhao, Y.; Procel, P.; Han, C.; Mazzarella, L.; Yang, G.; Weeber, A.; Zeman, M.; Isabella, O. Design and Optimization of Hole Collectors Based on Nc-SiOx:H for High-Efficiency Silicon Heterojunction Solar Cells. *Sol. Energy Mater. Sol. Cells* **2021**, 219, 110779. https://doi.org/10.1016/j.solmat.2020.110779.

(2) Zhao, Y.; Mazzarella, L.; Procel, P.; Han, C.; Tichelaar, F. D.; Yang, G.; Weeber, A.; Zeman, M.; Isabella, O. Ultra-Thin Electron Collectors Based on Nc-Si:H for High-Efficiency Silicon Heterojunction Solar Cells. *Prog. Photovoltaics Res. Appl.* **2022**, *30* (8), 809–822. https://doi.org/10.1002/pip.3502.

(3) Deligiannis, D.; Marioleas, V.; Vasudevan, R.; Visser, C. C. G.; Van Swaaij, R. A. C. M. M.; Zeman, M. Understanding the Thickness-Dependent Effective Lifetime of Crystalline Silicon Passivated with a Thin Layer of Intrinsic Hydrogenated Amorphous Silicon Using a Nanometer-Accurate Wet-Etching Method. *J. Appl. Phys.* **2016**, *119* (23), 235307. https://doi.org/10.1063/1.4954069.

(4) Bhattacharya, E.; Mahan, A. H. Microstructure and the Light-Induced Metastability in Hydrogenated Amorphous Silicon. *Appl. Phys. Lett.* **1988**, *52* (19), 1587–1589. https://doi.org/10.1063/1.99089.

(5) Langford, A. A.; Fleet, M. L.; Nelson, B. P.; Lanford, W. A.; Maley, N. Infrared Absorption Strength and Hydrogen Content of Hydrogenated Amorphous Silicon. *Phys. Rev. B* **1992**, *45* (23), 13367–13377. https://doi.org/10.1103/PhysRevB.45.13367.

(6) Santbergen, R.; Meguro, T.; Suezaki, T.; Koizumi, G.; Yamamoto, K.; Zeman, M. GenPro4 Optical Model for Solar Cell Simulation and Its Application to Multijunction Solar Cells. *IEEE J. Photovolt.* **2017**, *7* (3), 919–926. https://doi.org/10.1109/JPHOTOV.2017.2669640.

(7) Green, M. A.; Keevers, M. J. Optical Properties of Intrinsic Silicon at 300 K. *Prog. Photovoltaics Res. Appl.* **1995**, *3* (3), 189–192. https://doi.org/10.1002/PIP.4670030303.

(8) Johnson, P. B.; Christy, R. W. Optical Constants of the Noble Metals. *Phys. Rev. B* **1972**, *6* (12), 4370. https://doi.org/10.1103/PhysRevB.6.4370.

(9) Han, C.; Yang, G.; Procel, P.; O'Connor, D.; Zhao, Y.; Gopalakrishnan, A.; Zhang, X.; Zeman, M.; Mazzarella, L.; Isabella, O. Controllable Simultaneous Bifacial Cu‐Plating for High‐Efficiency Crystalline Silicon Solar Cells. *Solar RRL* **2022**, *6* (6), 2100810. https://doi.org/10.1002/solr.202100810.

(10) Hermle, M.; Feldmann, F.; Bivour, M.; Goldschmidt, J.C.; Glunz S.W. Passivating contacts and tandem concepts: Approaches for the highest silicon-based solar cell efficiencies, *Appl. Phys. Rev.* **2020**, *7*, 21305. https://doi.org/10.1063/1.5139202.
